# Fecal Viral Community Responses to High-Fat Diet in Mice

**DOI:** 10.1128/mSphere.00833-19

**Published:** 2020-02-26

**Authors:** Anjelique Schulfer, Tasha M. Santiago-Rodriguez, Melissa Ly, Joshua M. Borin, Jessica Chopyk, Martin J. Blaser, David T. Pride

**Affiliations:** a New York University, New York, New York, USA; b Department of Pathology, University of California, San Diego, California, USA; c Division of Biological Sciences, University of California, San Diego, California, USA; d Center for Advanced Biotechnology and Medicine, Rutgers University, Piscataway, New Jersey, USA; e Department of Medicine, University of California, San Diego, California, USA; University of Michigan-Ann Arbor

**Keywords:** virome, gut, microbiome, 16S rRNA, antibiotic perturbations, antibiotics, metabolism, high-fat diet

## Abstract

Alterations in diet can have significant impact on the host, with high-fat diet (HFD) leading to obesity, diabetes, and inflammation of the gut. Although membership and abundances in gut bacterial communities are strongly influenced by diet, substantially less is known about how viral communities respond to dietary changes. Examining fecal contents of mice as the mice were transitioned from normal chow to HFD, we found significant changes in the relative abundances and the diversity in the gut of bacteria and their viruses. Alpha diversity of the bacterial community was significantly diminished in response to the diet change but did not change significantly in the viral community. However, the diet shift significantly impacted the beta diversity in both the bacterial and viral communities. There was a significant shift away from the relatively abundant *Siphoviridae* accompanied by increases in bacteriophages from the *Microviridae* family. The proportion of identified bacteriophage structural genes significantly decreased after the transition to HFD, with a conserved loss of integrase genes in all four experimental groups. In total, this study provides evidence for substantial changes in the intestinal virome disproportionate to bacterial changes, and with alterations in putative viral lifestyles related to chromosomal integration as a result of shift to HFD.

**IMPORTANCE** Prior studies have shown that high-fat diet (HFD) can have profound effects on the gastrointestinal (GI) tract microbiome and also demonstrate that bacteria in the GI tract can affect metabolism and lean/obese phenotypes. We investigated whether the composition of viral communities that also inhabit the GI tract are affected by shifts from normal to HFD. We found significant and reproducible shifts in the content of GI tract viromes after the transition to HFD. The differences observed in virome community membership and their associated gene content suggest that these altered viral communities are populated by viruses that are more virulent toward their host bacteria. Because HFD also are associated with significant shifts in GI tract bacterial communities, we believe that the shifts in the viral community may serve to drive the changes that occur in associated bacterial communities.

## Abstract (precis)

Prior studies have shown that high-fat diet (HFD) can have profound effects on the gastrointestinal (GI) tract microbiome and also demonstrate that bacteria in the GI tract can affect metabolism and lean/obese phenotypes. We investigated whether the composition of viral communities that also inhabit the GI tract are affected by shifts from normal to HFD. We found significant and reproducible shifts in the content of GI tract viromes after the transition to HFD. The differences observed in virome community membership and their associated gene content suggest that these altered viral communities are populated by viruses that are more virulent toward their host bacteria. Because HFD also are associated with significant shifts in GI tract bacterial communities, we believe that the shifts in the viral community may serve to drive the changes that occur in associated bacterial communities.

## INTRODUCTION

Obesity is a problem worldwide, and much of the epidemic in the Western world is related to dietary excess. The consumption of diets high in fat content can be directly correlated with obesity (1–3) and its associated conditions, including diabetes (4), coronary artery disease, obstructive sleep apnea, and fatty liver (5–7). More recent work has demonstrated that the vast communities of microbes inhabiting human body surfaces (8), collectively known as the microbiome, also are affected by alterations in diet; these communities characteristically change in response to high-fat diets (HFD) (9–11). The organisms forming the bacterial biota in the human gastrointestinal (GI) tract have been the primary microbes studied in response to diet changes, and their responses to HFD suggest that they may play central roles in the metabolic changes that result in both obesity and diabetes (12–15).

Our body surfaces also are inhabited by robust communities of viruses (16–20), many of which are bacteriophages, whose role and responses to shifts in diet have not been well characterized (16, 21). The relationships of bacteriophages and their prokaryotic hosts may be antagonistic (killing their hosts) or mutualistic (integrating into host genomes and potentially providing beneficial gene functions) (22). Because these viral communities have the capacity to alter the resident bacterial biota, they could play important roles in shaping cellular microbiomes.

The study of the human virome lags considerably behind that of the bacteriome. Particular viruses may persist in the human gut virome, with characteristics reflecting the diet of the human host (16). This relationship may primarily represent changes in gut bacteria and with their associated phages (16). In mice, antibiotic administration increases the reservoir for putative antibiotic resistance genes in the virome (23). Gut bacteria also respond to bacteriophage-mediated perturbations (24). There is an approximate 20:1 ratio of virus particles to bacterial cells in the gut mucosa, which reflects the relationship of most of these phages with their host bacteria but may also reflect the ability of some bacteriophages to bind to mucosal layers in the gut (25). Binding of phages to mucosal layers serves as one type of *de facto* immune system, protecting the host from susceptible invading bacteria (25). Phages bound to mucosal layers may have broader host ranges than can be observed using plaque assays (26), reflecting their putatively broad immune functions based on their parasitism. As with humans (16), mouse gut viromes also respond to diet changes, with temperate phages that are associated with phylum *Bacteroidetes* becoming more prominent in mice receiving HFD (21).

The gut epithelial barrier may become impaired in mice receiving HFD (27, 28), due to reduced tight junction protein expression (29), with increased gut permeability, translocation of bacterial lipopolysaccharides into the bloodstream, and increased intestinal inflammation in humans (4, 30) and in mice (31). HFD also have been associated with downregulation of immunoglobulin synthesis and of components of the major histocompatibility complex, which may have further downstream immune effects (32).

Previously, we have studied the effects of exposure to low-dose penicillin during maturation and determined that it has long-term metabolic effects in mice (33). These mice also develop enhanced insulin resistance and liver disease when fed HFD (34). The effects of low-dose penicillin G appear to be microbiota related, as phenotypes associated with the microbes are transferable to germfree mice (35). In this study, we utilized the low-dose penicillin G model from our prior studies to characterize the effects of HFD on gut microbiota over 28 weeks (36). Our goals were to identify changes in the taxonomy of both gut bacterial and viral communities in response to HFD and to characterize the overall virome community composition and structure in response to diet changes.

## RESULTS

### Experimental design and G+C content differences.

**FIG 1:**
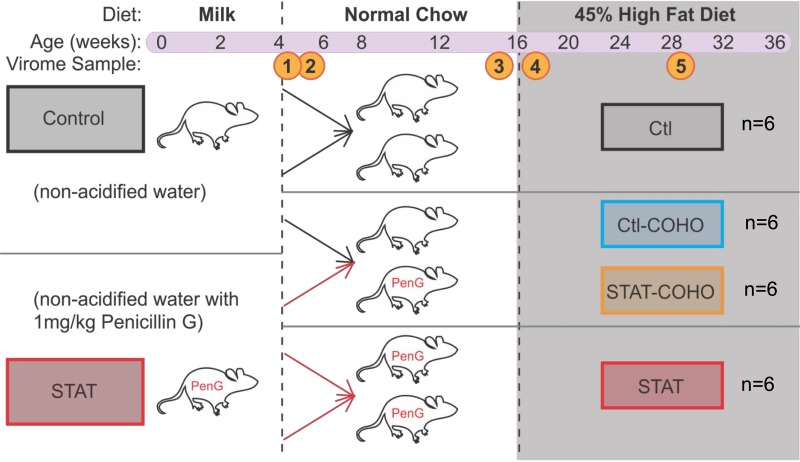
Study design. Litters were exposed (STAT) or not (control [CTL]) to low-dose penicillin G until weaning at day 28 of life. At day 32 (between the week 4 and 5 time points) of life, mice were litter-mixed and either cohoused (COHO; 3 STAT:3 CTL) or not (*n* = 6 mice per treatment group). Mice were given normal chow from weaning until week 16 and then switched to a 45% high-fat diet (HFD). Fecal samples and scale weight were collected regularly throughout the experiment. Samples were collected at week 4 (1), week 5 (2), week 15 (3), week 16 (4), and week 28 (5) of life, and fecal samples were pooled for 6 female mice in each group at each time point for further microbiome assessments.

We exposed C57BL/6J pregnant female mice to low-dose penicillin G (subtherapeutic antibiotic treatment [STAT]) in their drinking water (Fig. 1), which continued until their pups were 28 days old and had been weaned (33, 35, 36). Control mice and their pups were exposed in parallel to untreated water without antibiotics (CTL). To study the transmissible factors of the microbiota, pups were litter-mixed at day 32 of life and half of the CTL and half of the STAT group were cohoused at a 1:1 ratio to create 4 distinct groups (36). The CTL group (*n* = 6) consisted of mice that had only been housed with other CTL mice. The STAT group (*n* = 6) consisted of mice that had been housed only with other STAT mice. The CTL-COHO group (*n* = 6) was composed of mice that had never been exposed to penicillin G and then were cohoused with mice that had received penicillin G. The STAT-COHO group (*n* = 6) consisted of mice that had received penicillin G until day 28 of life and then were cohoused with CTL mice. All mice remained with these cagemates for the duration of the study. Fecal samples were collected and combined from the same six females per group at weaning (week 4, sample 1) and 5 days after cohousing (week 5, sample 2). All groups were exposed to normal diets from weeks 4 to 15 and then were transitioned to a 45% high-fat diet (HFD) from weeks 16 to 36. Fecal samples were collected just before (week 15, sample 3) and just after (week 16, sample 4) transition to the HFD. The final sample was collected at week 28 of life (sample 5) after the mice had consumed HFD for 12 weeks (Fig. 1). Feces from each sample (samples 1 to 5) were pooled for each of the four treatment groups (CTL, STAT, CTL-COHO, and STAT-COHO), yielding 20 total samples.

We isolated the viromes from feces of each group of mice, as described previously (17), which involved sequential filtering to remove cellular debris, CsCl density gradient ultracentrifugation, and DNA extraction from intact virions. Resulting DNA was sequenced using semiconductor sequencing (37) for a total of 10,590,368 reads after quality filtering, with a mean length of 216 nucleotides. We sequenced ∼2.65 million reads per group with ∼0.53 million reads per time point collected. The average G+C content for all the quality reads was 42.3% overall; however, there were significant differences (*P* = 0.009, *t* test) in G+C contents of viromes from mice receiving normal diets (40.8%) compared to those from mice on HFD (44.5%) (see Fig. S1 in the supplemental material). These differences suggested a shift in the composition of the viral community with the shift to HFD.

10.1128/mSphere.00833-19.1 FIG S1 Percent GC content (±standard error) among the virome reads recovered from mice across time points and diets. The *y* axis represents the percent GC content. The *P* value is shown above the bars and was determined by comparing the means between mice on HFD versus normal chow using a two-tailed *t* test. Download FIG S1, PDF file, 0.03 MB. Copyright © 2020 Schulfer et al. 2020 Schulfer et al. This content is distributed under the terms of the Creative Commons Attribution 4.0 International license.

### Changes in alpha diversity after transitioning to HFD.

**FIG 2:**
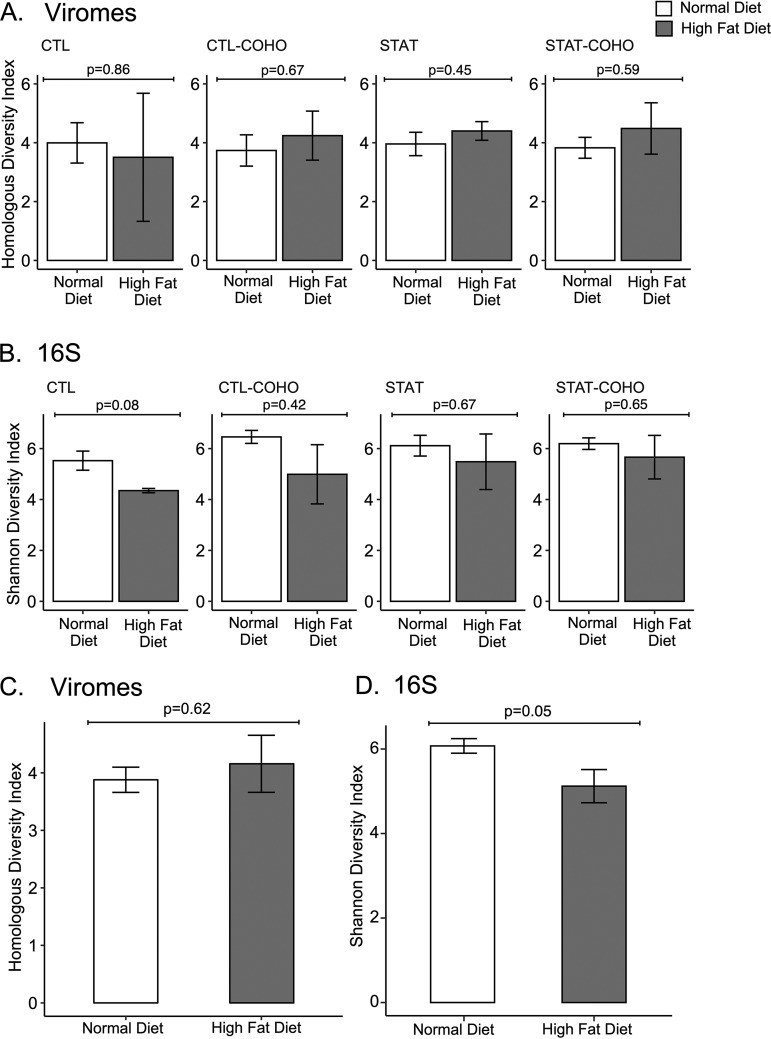
Homologous virus diversity indices (±SE) among the viral communities and Shannon diversity indices (±SE) among the bacterial biota for mice receiving normal chow or HFD. Panels A and B show the viral diversity (A) or bacterial diversity (B) with mice grouped based on their treatment status: penicillin G (STAT), control mice (CTL), STAT mice cohoused with CTL mice (STAT-COHO), and CTL mice cohoused with STAT mice (CTL-COHO). Panels C and D show the viral diversity (C) and bacterial diversity (D) with mice grouped by diet type only. *P* values represent comparisons between different diet types using a two-tailed *t* test.

**FIG 3:**
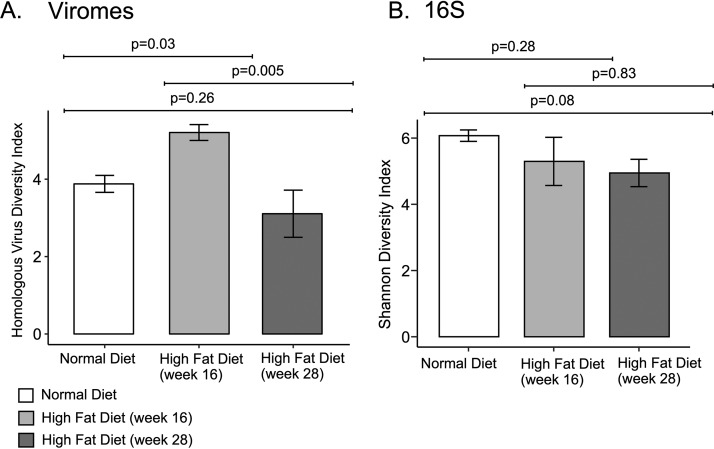
Homologous virus diversity indices (±SE) among viral communities (A) and Shannon diversity indices (±SE) among bacterial communities (B) in mice on normal chow and with HFD separated by week. *P* values represent comparisons between different diet groups using ANOVA.

We assembled virome reads from each time point to construct larger viral contigs to improve searchability for sequence similarities. For each sample, we assembled a mean of 3,572 ± 1,497 contigs, with 94.9% ± 1.8% of the total sequence reads assembling into viral contigs (Table S1). We first examined the alpha diversity of both the viral and bacterial communities. The alpha diversity for both the viral and bacterial communities was not significantly different based on STAT and cohousing status both when mice were fed normal chow compared to an HFD (*P* > 0.05, analysis of variance [ANOVA]). Additionally, when mice were separated into their respective STAT and cohousing status groups, there was no significant differences in either the bacterial or viral diversity based on diet (*P* > 0.05, *t* test [Fig. 2A and B]). When grouping mice regardless of STAT and cohousing status, we found that after the transition from normal chow to HFD, there was a nonsignificant increase in the alpha diversity of the viral communities (Fig. 2C). In contrast, in the bacterial communities, alpha diversity was significantly reduced after the transition to HFD (Fig. 2D), consistent with prior studies (38). We further parsed the alpha diversity by comparing each time point following transition from normal chow to HFD (Fig. 3A). In the short term, we identified a significant increase in alpha diversity in the viromes after the transition to HFD (*P* = 0.03, ANOVA); however, this trend in alpha diversity was significantly reduced by week 28 (*P* = 0.005, ANOVA) (Fig. 3A). Together, these data indicate that the community richness of the virome did not passively follow the bacteriome after the dietary transition to HFD.

10.1128/mSphere.00833-19.7 TABLE S1 Virome reads and assembly statistics. Download Table S1, DOCX file, 0.02 MB. Copyright © 2020 Schulfer et al. 2020 Schulfer et al. This content is distributed under the terms of the Creative Commons Attribution 4.0 International license.

### Beta diversity in response to high-fat diets.

**FIG 4:**
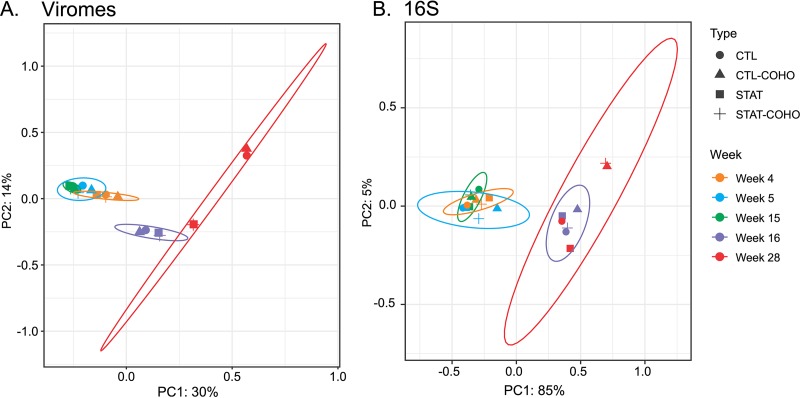
Principal-coordinate analysis representations of beta diversity based on Jaccard distances for viral communities (A) and weighted Unifrac distances for bacterial communities (B) of mice that were exposed to penicillin G (STAT) or not (CTL) and cohoused mice that were exposed to penicillin G (STAT-COHO) or not (CTL-COHO). All mice received normal diets at weeks 4, 5, and 15 and were switched to HFD at weeks 16 and 28. PERMANOVA was performed using the Adonis function (model formula ∼ diet × time) and (model formula ∼ STAT) to determine statistical differences. Ellipses are drawn at 95% confidence intervals for week sampled.

We next examined the beta diversity among the viral communities across the dietary transition. We found that viral communities clearly differed before and after the transition in diet (*P* = 0.001 and *R*^2^ = 0.26, Adonis [Fig. 4A]). The viral communities of mice were also distinct based on the time point from which they were derived (*P* = 0.006 and *R*^2^ = 0.09, Adonis). A similar trend in response to HFD also was observed in bacterial communities, with two distinct groups based on diet (*P* = 0.001 and *R*^2^ = 0.83, Adonis [Fig. 4B]). However, there was no significant demarcation due to time (*P* = 0.31, Adonis). For both bacterial and viral communities, there was no significant difference based on STAT and cohousing status.

To determine whether there were significant differences in the numbers of homologous viruses shared between different groups of mice, we used permutation testing (Table 1), as we have described previously (39, 40). There was a significant conservation of viromes in mice receiving normal diets (*P* = 0.02), but none among the mice receiving HFD (*P* = 0.32). We also examined whether time might be a factor in the conservation of virome contents and found that the only significant conservation of virome contents occurred in the mice receiving HFD at week 28 (*P* = 0.03). There were no significant distinctions between the viromes of the mice based on their cohousing status. In total, these results indicate that the beta diversity of both the viral and bacterial communities was affected by the transition to HFD, with lesser effects of age and antibiotic or cohousing exposure status. There was no consistent variation in the viromes to indicate a link between mouse exposure to penicillin G early in life and the fecal virome compositions (Fig. 4 and Table 1).

**TABLE 1:** Viral homologues across time, diet, antibiotic, and cohousing

Grouping	% homologous within group^*a*^	% homologous between groups^*a*^	*P* value^*b*^
By diet			
Normal chow	74.11 ± 9.54	43.59 ± 16.8	**0.02**
HFD	50.00 ± 18.96	42.91 ± 17.24	0.32
By time			
4 wks (normal chow)	42.11 ± 10.40	32.00 ± 14.16	0.31
5 wks (normal chow)	58.07 ± 8.24	40.07 ± 18.38	0.18
15 wks (normal chow)	53.89 ± 9.56	35.39 ± 17.50	0.19
16 wks (HFD)	33.43 ± 10.40	26.95 ± 8.44	0.37
28 wks (HFD)	33.80 ± 9.87	17.48 ± 5.38	**0.03**
By cohousing			
No cohousing	57.61 ± 20.11	56.28 ± 21.27	0.41
Cohousing	50.03 ± 18.72	55.93± 21.42	0.61
By penicillin G exposure			
No penicillin	51.96 ± 25.64	54.68 ± 19.27	0.52
Penicillin	59.33 ± 16.99	54.15 ± 19.34	0.45

a Based on the mean of 10,000 iterations. A total of 1,000 random contigs were sampled per iteration.

b *P* value based on the fraction of times the estimated percent homologous contigs for each group exceeded that between groups. Bold text denote significant *P* values (*P* < 0.05).

### Gene content differences in viromes in response to diet.

Using BLASTX to identify virome contigs that had homologies to known viral genes in the NCBI Non-redundant (NR) database, we identified substantial numbers of homologues (Fig. S2). We found that 84% ± 9% of the reads were assembled into contigs with homologies to known viruses, 6% ± 3% were homologous to bacteria, and 10% ± 9% had no known homologies. It is important to note that there were bacteriophages with putative lysogenic lifestyles present in these viromes, which can result in homologies to bacterial genes in virome studies (17). However, there were no significant differences between the proportions of reads in contigs that were homologous to known viruses in the samples based on diet (83% in normal chow and 86% in HFD).

10.1128/mSphere.00833-19.2 FIG S2 Percentages of virome reads belonging to contigs with significant sequence similarities within the NCBI NR database. The percentage of reads was determined based on the raw number of reads used to assemble each contig. The percentage of reads is shown on the *y* axis. Download FIG S2, PDF file, 0.03 MB. Copyright © 2020 Schulfer et al. 2020 Schulfer et al. This content is distributed under the terms of the Creative Commons Attribution 4.0 International license.

**FIG 5:**
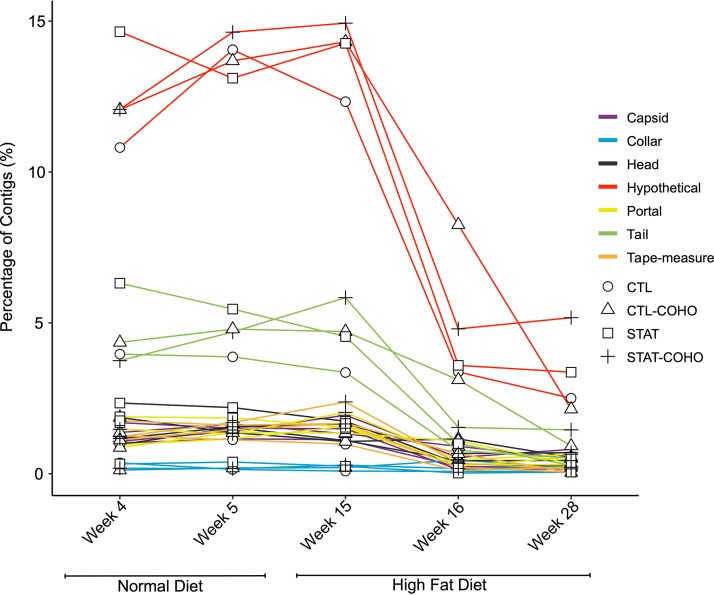
Assignment of virome contigs with homologues to structural genes in the viromes of mice, according to time point and diet type. The *y* axis represents the percentage of putative structural genes identified. Shape represents the different treatment groups: STAT, CTL, STAT-COHO, and CTL-COHO.

**FIG 6:**
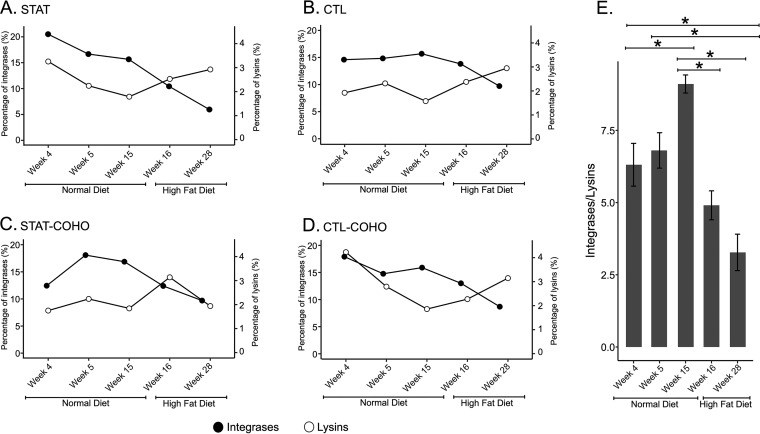
Assignment of virome contigs with homologues to integrases or lysin genes in the viromes of mice by time point and diet type. For panels A to D, the *y* axes represent the percentage of contigs with integrase homologues, the *z* axes represent the percentage of contigs with lysin homologues, and the *x* axes represent the different time points measured in the mice. The difference in the abundances of integrases in mice on normal diets and HFD was statistically significant (*P* < 0.05) by two-tailed *t* test. For panel E, the *y* axis represents the ratio of integrase genes to lysin genes (±SE) for all antibiotic exposures. *, values are statistically significant (*P* ≤ 0.05) by ANOVA.

Next, we characterized the viral homologues according to function. The proportion of each of the phage structural genes, aside from the Collar, was significantly lower after the transition from normal chow to HFD (*P* < 0.001, *t* test [Fig. 5]). In addition, similar to what was observed previously, the proportion of phage structural genes was not significantly different based on STAT and cohousing status (*P* > 0.05, ANOVA [Fig. 5]). We also identified nonsignificant increases in the proportions of lysins (*P* = 0.23, *t* test) and concomitant, significant decreases in integrases (*P* = 0.0002, *t* test) associated with the transition to HFD (Fig. 6). Again, we found no significant difference in the proportion of integrases or lysins based on STAT and cohousing status (*P* > 0.05, ANOVA [Fig. 5]). Moreover, we found that regardless of antibiotic exposure, the ratio of integrase to lysin genes was significantly lower at week 28 than at all weeks on the normal diet (*P* < 0.05, ANOVA [Fig. 6E]). These data suggest a shift in the types of viruses, according to lifestyle and host (bacterial) interactions.

### Shifts in virome contents.

**FIG 7:**
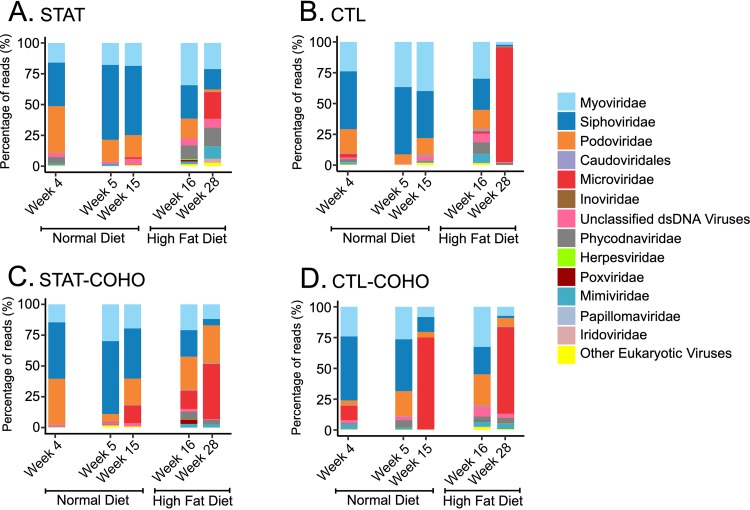
Proportion of reads assigned to viral contigs with TBLASTX hits to the specified virus families. The *y* axis represents the percentage of reads assigned to contigs homologous to each family.

**FIG 8:**
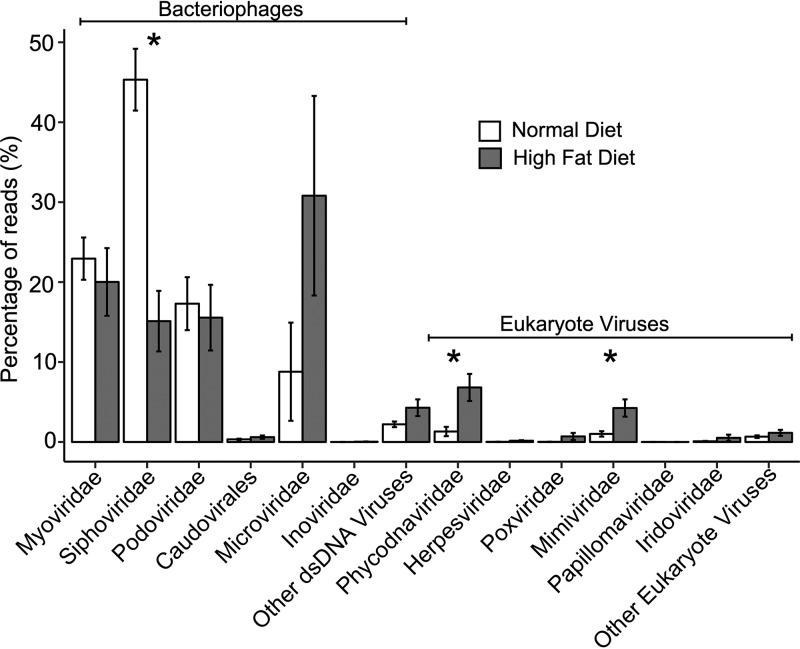
Proportion of virome reads (±SE) belonging to contigs with TBLASTX hits to the specified virus families in all mice receiving normal chow or receiving HFD. The *y* axis represents the percentage of reads assigned to contigs homologous to each family shown on the *x* axis. *, values are statistically significant (*P* ≤ 0.05) using a two-tailed *t* test.

We next examined the types of viruses present in the fecal viral communities. Using TBLASTX analysis, we profiled the optimal matches for each virome contig and categorized each by virus family. We weighted the results based on contig read coverage to provide additional representation to those contigs assembled from greater numbers of reads in each fecal virome. For the mice on normal chow, most contigs were homologous to caudoviruses, including the families *Siphoviridae*, *Myoviridae*, and *Podoviridae* (Fig. 7). However, with change to HFD, the relative proportions of caudoviruses decreased, while the proportions of phages from the family *Microviridae* increased substantially by week 28. There was little variation in the profiles that could be observed regardless of whether the mice had penicillin G exposure (STAT [Fig. 7A]) or not (CTL [Fig. 7B]) or were cohoused with mice that had been exposed to penicillin G (STAT-COHO [Fig. 7C]) or not (CTL-COHO [Fig. 7D]). To search for a more subtle signal, we combined the viromes from all mice on normal diets and all mice on HFD. We observed a significant reduction in the representation of *Siphoviridae* after the change to HFD and a significant increase in the representation of eukaryotic viruses *Phycodnaviridae* and *Mimivirdae* (*P* < 0.05, *t* test [Fig. 8]). Additionally, when grouping the HFD samples by week, the *Siphoviridae* decreased significantly at both week 16 and week 28 compared to the normal chow samples, while the proportion of *Phycodnaviridae* was only significantly higher at week 16 with the HFD and that of *Mimivirdae* only significantly higher at week 28 compared to that with normal chow (*P* < 0.05, ANOVA [Fig. S3]). Using a Pearson correlation test, we also determined that the relative abundance of *Siphoviridae* (*R* = 0.69 and *P* = 0.0008, Pearson) correlated positively and those of *Microviridae* (*R* = −0.44 and *P* = 0.049, Pearson), *Mimiviridae* (*R* = −0.52 and *P* = 0.02, Pearson), *Phycodnaviridae* (*R* = −0.57 and *P* = 0.009, Pearson), and *Iridoviridae* (*R* = −0.45 and *P* = 0.047, Pearson) correlated negatively with the percentage of integrase homologues.

10.1128/mSphere.00833-19.3 FIG S3 Proportion of virome reads (±standard error) belonging to contigs with TBLASTX hits to the specified virus families in all mice receiving normal chow (white bars) and mice receiving HFD separated by week (light gray, week 16; dark gray, week 28). The *y* axis represents the percentage of reads assigned to contigs homologous to each family. *, values are statistically significant (*P* ≤ 0.05) by ANOVA. Download FIG S3, PDF file, 0.03 MB. Copyright © 2020 Schulfer et al. 2020 Schulfer et al. This content is distributed under the terms of the Creative Commons Attribution 4.0 International license.

Characterizing the viral communities by quantifying the relative number of contigs assigned to specific viral families rather than the number of reads belonging to contigs that were homologous to specific virus families, we identified similarly significant trends (Fig. S4 to S6). Specifically, we found a significant decrease in the proportion of contigs assigned as *Siphoviridae* when the mice were transitioned to HFD. While we identified large numbers of reads assigned to the family *Microviridae* (Fig. 7), especially after transition to HFD, only a small proportion of contigs were assigned to this family (Fig. S4 to S6). These results indicate that there were relatively few highly abundant *Microviridae* family members. We also identified a greater proportion of contigs representing eukaryotic viruses, including *Phycodnaviridae*, *Herpesviridae*, and *Poxviridae*, in the mice after the transition to HFD (*P* < 0.05, *t* test [Fig. S5]). Although none of these viruses were highly represented according to read abundances (Fig. 7 and 8), the profiles of virus families changed significantly after the transition to HFD, regardless of the analytical methodology. We found similar, significant trends when we evaluated the proportion of contigs assembled from reads that were normalized to a minimum sampling depth (Fig. S5B and S6B).

10.1128/mSphere.00833-19.4 FIG S4 Viral contigs with TBLASTX hits to specified virus families. The *y* axis represents the percentage of contigs homologous to each family. Control mice are designated CTL, mice that were exposed to penicillin G are designated STAT, CTL-COHO mice were control mice cohoused with STAT mice, and STAT-COHO mice were STAT mice cohoused with CTL mice. Download FIG S4, PDF file, 0.03 MB. Copyright © 2020 Schulfer et al. 2020 Schulfer et al. This content is distributed under the terms of the Creative Commons Attribution 4.0 International license.

10.1128/mSphere.00833-19.5 FIG S5 Viral contigs with TBLASTX hits to the specified virus families in mice receiving normal chow (white bars) and mice receiving HFD (gray). In panel A, the *y* axis represents the percentage of contigs (±standard error) homologous to each family. In panel B, the *y* axis represents the percentage of contigs (±standard error) homologous to each family, with the number of reads normalized to the smallest sampling depth. *, values are statistically significant (*P* ≤ 0.05) using a two-tailed *t* test. Download FIG S5, PDF file, 0.03 MB. Copyright © 2020 Schulfer et al. 2020 Schulfer et al. This content is distributed under the terms of the Creative Commons Attribution 4.0 International license.

10.1128/mSphere.00833-19.6 FIG S6 Viral contigs with TBLASTX hits to the specified virus families in mice receiving normal chow (white bars) and mice receiving HFD separated by week (light gray, week 16; dark gray, week 28). In panel A, the *y* axis represents the percentage of contigs (±standard error) homologous to each family. In panel B, the *y* axis represents the percentage of contigs (±standard error) homologous to each family, with the number of reads normalized to the smallest sampling depth. *, values are statistically significant (*P* ≤ 0.05) using ANOVA. Download FIG S6, PDF file, 0.04 MB. Copyright © 2020 Schulfer et al. 2020 Schulfer et al. This content is distributed under the terms of the Creative Commons Attribution 4.0 International license.

### Changes in bacterial communities.

**FIG 9:**
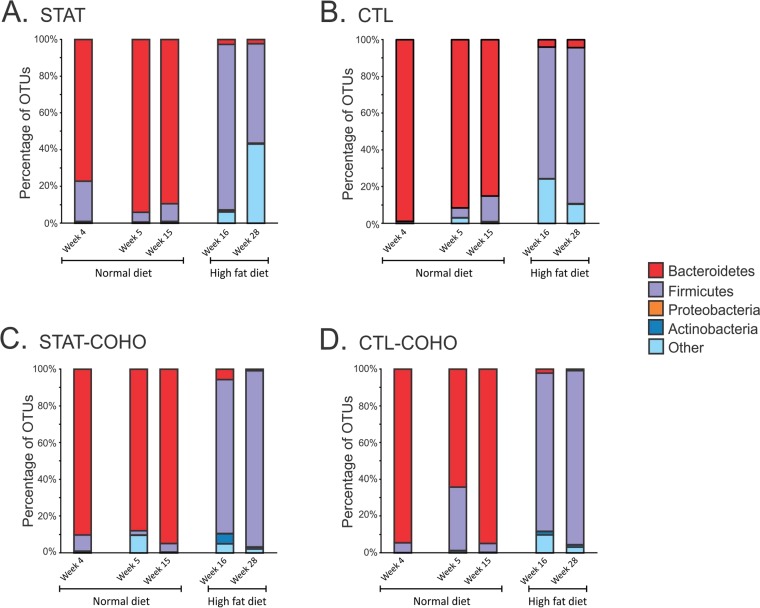
Bacterial taxonomy based on 16S rRNA sequences identified at the phylum level. The *y* axis represents the percentage of operational taxonomic units (OTUs) assigned to each phylum. The difference in the representation of the phyla *Bacteroidetes* and *Firmicutes* in mice on normal diets and HFD was statistically significant (*P* < 0.001) by two-tailed *t* test for each group.

To determine whether shifts in virome contents followed changes in the compositions of the bacterial community, we characterized the fecal bacterial biota. Sequencing of the V1-V2 segment of 16S rRNA across the 20 samples showed that there were a total of 2,486,606 reads after quality filtering, with a mean length of 340 nucleotides. In total, there were ∼497,321 reads per group, with ∼124,330 reads per time point. The phylum *Bacteroidetes* dominated the bacterial community when the mice were fed normal chow (weeks 4 to 15), while *Firmicutes* predominated after the mice were begun on HFD (weeks 16 to 28), a shift that was statistically significant (*P* < 0.001) and was apparent regardless of cohousing status (Fig. 9). These data indicate that HFD also had significant and profound impacts on the fecal bacterial community, suggesting that the changes observed in viromes (Fig. 7) and the bacterial communities (Fig. 9) could be interrelated.

## DISCUSSION

While it has been well described that changes in diet significantly affect gut bacterial communities (41, 42), substantially less is known about how viral communities respond. Some members of the human gut viral community are highly individual specific, appear remarkably stable over time, and have similar ecologies to one another based on diet (16). In mice, luminal and mucosal viral communities differ, reflecting the associated bacterial biota in the GI tract, but HFD consumption resulted in greater numbers of temperate bacteriophages in the mucosa than in the lumen (21). Transitioning mice to HFD to allow direct comparisons of gut virome contents in the same mice before and after consuming HFD allowed us to examine this issue more precisely. The decreases in the proportions of all 3 major caudovirus families (*Myoviridae*, *Siphoviridae*, and *Podoviridae*) after the transition to HFD, regardless of analytical method (Fig. 8 and Fig. S3 to S^6^), were consistent. However, in contrast to the previous research on mucosal and luminal viromes of mice fed an HFD (21), we identified significant decreases in the proportion of integrases, suggesting a reduction in the overall numbers of temperate bacteriophages. Indeed, bacteriophages from the family *Siphoviridae*, which often have lysogenic lifestyles (43, 44), were significantly reduced on HFD. This phage phenotype transition may have consequences for gut health, as a proper balance between lysis and lysogeny is suggested to maintain a healthy microbial community (45). Specifically, improper balance between these lifestyles in the gut is associated with leukemic diseases (46) and inflammatory bowel disease (47).

The significant changes we observed in the viromes were accompanied by shifts in the bacterial communities in response to HFD. After the transition to HFD, the shift from *Bacteroidetes* to *Firmicutes* (Fig. 9) was accompanied by significant reductions in caudoviruses and increases in *Microviridae*, especially at week 28 (Fig. 7 and Fig. S3). A recent study has found that microviruses are persistent inhabitants of the human gut (48). However, because they have been identified in both *Bacteroidetes* and *Firmicutes* (49), the source of their increased representation in this study could not be determined. We did not identify greater numbers of microviruses in response to the diet change (Fig. S5 and S6), but we did find rather significant changes in their relative abundances (Fig. 7 and 8). It is uncertain whether the virome differences merely reflect changes in the bacterial communities or whether phages actively promoted shifts in the bacterial communities; the observed shifts could reflect the *Firmicute* predominance if these microviruses infect *Firmicutes*, or conversely, they could represent increased predation of the *Bacteroidetes* if *Bacteroidetes* are their hosts.

Early-life STAT has a substantial impact on murine phenotypes after transitioning to HFD (34, 36), which is likely associated with the composition of the gut bacterial community (35). Our data did not indicate that the antibiotics had any significant effects on the composition of the gut virome (Fig. 4). In this case, the change caused by the diet transition may have masked any substantial effects of the STAT on the virome. Moreover, after transitioning to HFD, alpha diversity of the bacterial biota was significantly reduced, as observed previously (50–52), but the viral diversity did not change significantly in the long term (Fig. 2 and 3). We have previously observed a parallel trend in the GI tract of human subjects receiving long-term antibiotics (53), where viral diversity was not significantly affected despite the significant reduction in bacterial diversity. In that study, we observed greater numbers of eukaryotic viruses that likely compensated for the reduction in bacteriophages. In this study, we also observed an increase in the number of eukaryotic viruses following the transition to HFD, suggesting commonalities in the effect of antibiotics and diet on virome community composition (Fig. S4 and S5).

While the association of *Herpesviridae* and *Poxviridae* with human disease is well understood, the potential impact of *Mimiviridae* and *Phycodnaviridae* on health is only now being recognized. The family *Mimiviridae* has been linked to clinical cases of pneumonia, and recent evidence suggests that *Phycodnaviridae* may affect substantially more than just algal species (54–56). For example, the inoculation of a *Phycodnaviridae* virus (ATCV-1) into the intestinal tracts of 9- to 11-week-old mice resulted in a decrease in cognitive performance (56). However, it is important to note that *Mimiviridae* (size, ∼0.7 μm) were likely unable to pass through the 0.22-μm filter used to isolate the viral community in this study. Therefore, the *Mimiviridae* homology identified here was likely due to smaller unknown viruses, such as virophage, carrying *Mimiviridae*-like elements (57, 58).

The beta diversity shifts in viromes and the bacterial microbiome were most pronounced between weeks 15 and 16 when the mice were transitioned to HFD (Fig. 4). While a few studies have characterized murine GI tract viromes (21, 24, 59, 60), none have observed shifts in bacterial and viral ecology in the absence of perturbations such as dietary shifts. We do not believe the shift in ecology between weeks 15 and 16 is due to age (61); it more likely represents a direct response to HFD. While there was a clear and significant distinction between the viral communities observed across the specific time points (Fig. 4A), none was observed within the bacterial communities (Fig. 4B). It is possible that these differences reflect methodologic variation (shotgun metagenomics for viruses versus 16S rRNA amplicon sequencing for bacteria).

The virome changes we observed after the transition to HFD included reductions in *Siphoviridae* accompanied by gains in others such as *Microviridae* and several eukaryotic viruses (Fig. 8 and Fig. S3). However, it important to note that the amplification step (MDA) used in the production of the viromes is known to introduce biases (62, 63). The bacterial community changes included increases in *Firmicutes* with a concomitant decline in *Bacteroidetes* (Fig. 9). The reduction in integrase gene homologues found in the viral community of all four groups of mice (Fig. 6) after the start of the HFD is striking and is consistent with a global shift from a more temperate bacteriophage community to a community with more lytic phages. This phenotypic shift in the viral community was unanticipated but quite consistent, and it supports the hypothesis that the viral community plays a role in driving the significant changes observed in the bacterial communities and/or in maintaining these altered bacterial community phenotypes. In any event, exposure to HFD has led to this emergent property of the virome with a loss in abundance of integrase genes, which are markers for lytic versus lysogenic viral lifestyles.

Despite these novel findings, there were some limitations to our study. The long duration of our study limited our ability to reproduce the experiment multiple independent times. Because we observed no significant differences between STAT or cohousing status, these groups (the STAT and STAT-COHO groups and the CTL and CTL-COHO groups) may be considered replicates, with each group demonstrating similar, significant trends. Moreover, multiple confounding factors, such as genetic lineage and animal husbandry practices, may drive interlab variability in all aspects of the microbiome (64). Therefore, although these factors were controlled in this study, future studies should aim to ensure the generalizability and confirm the identities and relative abundances of the viral community members after the HFD transition in other genetic lineages.

## MATERIALS AND METHODS

### Animal experiments.

The protocol was approved by the New York University School of Medicine (NYUSoM) Institutional Animal Care and Use Committee (IACUC). The mice used in this experiment were part of a larger cohort described by Schulfer and colleagues (36). Male and female C57BL/6J (stock no. 000664) mice were received at 6 weeks of age from Jackson Laboratories and allowed to adjust to the NYUSoM animal facility for 1 week prior to breeding. After 5 days, breeding pairs were separated and pregnant dams were randomized into control (CTL) or subtherapeutic antibiotic treatment (STAT) groups. Penicillin G (6.8 mg/liter; STAT) was added or not (CTL) to dams’ drinking water at ∼ day 14 of gestation, as described previously (49). To maintain a fresh supply of penicillin G, water containers were changed twice weekly. Pups were weaned at day of life 28, at which point antibiotics were stopped in the STAT group. All mice had *ad libitum* access to water and chow (Purina Mills International Diet 5001; 4.07 kcal/g with 13.5% kcal from fat) and were maintained on a 12-h light/dark cycle. After removing dams from pups and stopping antibiotic treatment at day 28 of life, pups were moved to clean cages for 3 to 4 days to remove residual antibiotics from their environment. After this washout phase, mice were randomly assigned to new cages. Half of each STAT or CTL treatment group was placed with other mice of the same treatment, and half were cohoused at a 1:1 ratio of STAT to CTL mice. At week 16, all mice were switched to HFD (4.73 kcal/g with 45% kcal from fat; rodent diet D12451; Research Diets, New Brunswick, NJ). For each treatment group, 6 mice were individually placed into sterile containers and fresh fecal pellets were collected from the container and immediately frozen at −80 ˚C until use in this study. Fecal samples were collected at defined time points: week 4, week 5, week 15, week 16, and week 28. Mice continued on the treatment until week 36 as part of a larger cohort described by Schulfer and colleagues (36).

### Analysis of viromes.

Fecal viromes were prepared by diluting 0.4 g of feces in 4 ml of a sodium chloride, magnesium sulfate buffer (SM buffer) and vortexing for 40 min to separate viral particles, with spinning at 4,000 × *g* for 10 min to pellet the remaining solid material. The supernatants then were filtered sequentially using 0.45-μm and 0.2-μm filters (VWR) to remove cellular and other debris and then purified on a cesium chloride gradient according to previously described protocols (17). Only the fraction with a density corresponding to most known bacteriophages (65) was retained, further purified on Amicon YM-100 protein purification columns (Millipore, Inc.), treated with DNase I, and subjected to lysis and DNA purification using the Qiagen UltraSens virus kit (Qiagen). Recovered DNA was screened for the presence of contaminating bacterial nucleic acids by quantitative 16S rRNA gene PCR using primers 8F (AGAGTTTGATCCTGGCTCAG) and 357R (CTGCTGCCTYCCGTA) in Power SYBR green PCR master mix (Thermo Fisher Scientific). Viral DNA then was amplified using GenomiPhi Hy MDA amplification (GE Healthcare), fragmented to roughly 200 to 400 bp using a Bioruptor (Diagenode), and utilized as input to create libraries using the Ion Plus fragment library kit according to the manufacturer’s instructions. Libraries then were sequenced using 316 chips on an Ion Torrent personal genome machine. We trimmed sequence reads according to modified Phred scores of 0.5 using CLC Genomics Workbench 9.0, removed any low-complexity reads with ≥8 consecutive homopolymers, and removed any reads with substantial length variation (<150 nucleotides or >300 nucleotides) or ambiguous characters prior to further analysis. Each virome was screened for human nucleic acids using BLASTN analysis (E value < 10^−5^) against the human reference database available at ftp://ftp.ncbi.nlm.nih.gov/genomes/H_sapiens/. Any reads with significant sequence similarities to human sequences were removed prior to further analysis using Ion Assist (www.thepridelaboratory.org).

Prior to assembly, read statistics (e.g., quantity, GC content) were calculated via the CLC Genomics Workbench 9.0. Sequence reads were then assembled using CLC Genomics Workbench 9.0 based on 98% identity with a minimum of 50% read overlap, which were more stringent than criteria developed to discriminate between highly related viruses (66). Because the shortest reads were 50 nucleotides, the minimum tolerable overlap was 25 nucleotides, and the average overlap was no less than 100 nucleotides depending on the characteristics of each virome. The consensus sequence for each contig was constructed according to majority rule, and any contigs of <200 nucleotides or with ambiguous characters were removed prior to further analysis.

Virome contigs were annotated using BLASTX against the NCBI NR database with an E value cutoff value of 10^−5^. Specific viral sequences were identified using Ion Assist (www.thepridelaboratory.org) by parsing BLASTX results for known viral genes, including replication, structural, transposition, restriction/modification, hypothetical, and other genes previously found in viruses for which the E value was at least 10^−5^. Each individual virome contig was annotated using this technique; however, if the best hit for any portion of the contig was to a gene with no known function, lower-level hits were used as long as they had known function and still met the E value cutoff. Virus types were determined by parsing the virus families from the TBLASTX best hits of each viral contig with an E value of <10^−20^. Analysis of shared sequence similarities present in each virome was performed by creating custom BLAST databases for each virome, comparing each database with all other viromes using BLASTN analysis (E value < 10^−10^), and these compiled data were used to calculate Jaccard distances using Ion Assist (www.thepridelaboratory.org). These distances then were used as input for principal-coordinates analysis using QIIME (67). Alpha diversity was determined using the homologous virus diversity index (based on Shannon Diversity) as we have previously described (68, 69). Data were visualized using the R package ggplot2 ver. 3.1.0 (70).

### Statistical analysis of differences among viromes.

Statistically significant differences in gene categories (integrases and lysins), alpha diversity, relative abundances of viral families, and G+C content between HFD and normal chow groups were determined by two-tailed *t* tests. Differences in alpha diversity and the relative abundance of viral families with HFD grouped by week and the ratio of integrases and lysins by week were determined by analysis of variance (ANOVA) with *post hoc* Tukey HSD (honestly significant difference) test. To assess whether viromes had significant overlap within or between groups, we performed a permutation test using Ion Assist (www.thepridelaboratory.org) based on resampling (10,000 iterations). We previously have used this test to identify significant differences between viromes of individuals and different treatment groups (39, 40, 71). Briefly, we simulated the distribution of the fraction of shared virome homologues from 2 different groups within individual mice that were randomly chosen. For each set, we computed the summed fraction of shared homologues using 1,000 random contigs between and within different mice, and from these computed an empirical null distribution of our statistic of interest (the fraction of shared homologues). The simulated statistics within each mouse were referred to the null distribution of intermouse comparisons, and the *P* value was computed as the fraction of times the simulated statistic for the each exceeded the observed statistic. To perform statistical tests on viral beta diversities, represented in principal-coordinate analysis plots, we performed permutational multivariate analyses of variance (PERMANOVA) in the R Vegan package using the Adonis function with 999 permutations.

### Analysis of 16S rRNA.

Genomic DNA was prepared from the fecal pellets of each subject and time point using the QIAamp DNA Stool MINI kit (Qiagen). We amplified the bacterial 16S rRNA gene V1-V2 hypervariable region using the forward primer 8F (AGAGTTTGATCCTGGCTCAG) fused with the Ion Torrent adaptor A sequence and one of 70 unique 10-bp barcodes, and reverse primer 357R (CTGCTGCCTYCCGTA) fused with the Ion Torrent adaptor P1 from each donor and sample type (72). PCRs were performed using Platinum high-fidelity PCR SuperMix (Invitrogen) with the following cycling parameters: 94°C for 10 min, followed by 30 cycles of 94°C for 30 s, 53°C for 30 s, and 72°C for 30 s and a final elongation step of 72°C for 10 min. Resulting amplicons were purified on a 2% agarose gel stained with SYBR Safe (Invitrogen) using the MinElute PCR purification kit (Qiagen). Amplicons were further purified with Ampure XP beads (Beckman-Coulter), and molar equivalents were determined for each sample by quantifying the amplicons using PicoGreen (Invitrogen) using a plate reader. Samples were pooled into equimolar proportions and sequenced on 316 chips using an Ion Torrent PGM according to manufacturer’s instructions (Life Technologies) (37). Resulting sequence reads were removed from the analysis if they were <180 nucleotides or >500 nucleotides, had any barcode or primer errors, contained any ambiguous characters, or contained any stretch of >8 consecutive homopolymers. Sequences then were trimmed according to any site that had a Phred score of less than 15 (73). Sequences then were assigned to their respective samples based on a 10-nucleotide barcode sequence and were further processed to remove reads that were greater than 3 standard deviations from the mean read lengths in any specimen.

We sequenced a minimum of 10,000 reads from each sample and analyzed the sequence data using Quantitative Insights Into Microbial Ecology (QIIME 1.5) (67). Representative OTUs from each set were chosen at a minimum sequence identity of 97% using the QIIME script pick_otus_through_otu_table, which uses the Greengenes database (74). Principal-coordinate analysis was performed based on beta diversity using weighted UniFrac distances (75) via the QIIME script beta_diversity_through_plots. Alpha diversity using the Shannon diversity index (76) was determined using QIIME. Statistical differences in alpha diversity were determined by two-tailed *t* tests. For beta diversity, statistical differences were determined with PERMANOVA (999 permutations).

### Availability of data and material.

All sequences are available for download in the NCBI Sequence Read Archive under accession number PRJNA437977. Ion Assist software is available for download at www.thepridelaboratory.org and runs on Windows XP or higher.

## References

[1] Ley RE, Backhed F, Turnbaugh P, Lozupone CA, Knight RD, Gordon JI 2005 Obesity alters gut microbial ecology. Proc Natl Acad Sci U S A 102:11070–11075. doi:10.1073/pnas.0504978102. 16033867 PMC1176910

[2] Ley RE, Turnbaugh PJ, Klein S, Gordon JI 2006 Microbial ecology: human gut microbes associated with obesity. Nature 444:1022–1023. doi:10.1038/4441022a. 17183309

[3] Turnbaugh PJ, Ley RE, Mahowald MA, Magrini V, Mardis ER, Gordon JI 2006 An obesity-associated gut microbiome with increased capacity for energy harvest. Nature 444:1027–1031. doi:10.1038/nature05414. 17183312

[4] Qin J, Li Y, Cai Z, Li S, Zhu J, Zhang F, Liang S, Zhang W, Guan Y, Shen D, Peng Y, Zhang D, Jie Z, Wu W, Qin Y, Xue W, Li J, Han L, Lu D, Wu P, Dai Y, Sun X, Li Z, Tang A, Zhong S, Li X, Chen W, Xu R, Wang M, Feng Q, Gong M, Yu J, Zhang Y, Zhang M, Hansen T, Sanchez G, Raes J, Falony G, Okuda S, Almeida M, LeChatelier E, Renault P, Pons N, Batto J-M, Zhang Z, Chen H, Yang R, Zheng W, Li S, Yang H, Wang J, Ehrlich SD, Nielsen R, Pedersen O, Kristiansen K, Wang J 2012 A metagenome-wide association study of gut microbiota in type 2 diabetes. Nature 490:55–60. doi:10.1038/nature11450. 23023125

[5] Eckel RH, Grundy SM, Zimmet PZ 2005 The metabolic syndrome. Lancet 365:1415–1428. doi:10.1016/S0140-6736(05)66378-7. 15836891

[6] Somers VK, White DP, Amin R, Abraham WT, Costa F, Culebras A, Daniels S, Floras JS, Hunt CE, Olson LJ, Pickering TG, Russell R, Woo M, Young T 2008 Sleep apnea and cardiovascular disease: an American Heart Association/American College of Cardiology Foundation Scientific Statement from the American Heart Association Council for High Blood Pressure Research Professional Education Committee, Council on Clinical Cardiology, Stroke Council, and Council on Cardiovascular Nursing. J Am Coll Cardiol 52:686–717. doi:10.1016/j.jacc.2008.05.002. 18702977

[7] Perlemuter G, Bigorgne A, Cassard-Doulcier AM, Naveau S 2007 Nonalcoholic fatty liver disease: from pathogenesis to patient care. Nat Clin Pract Endocrinol Metab 3:458–469. doi:10.1038/ncpendmet0505. 17515890

[8] Costello EK, Lauber CL, Hamady M, Fierer N, Gordon JI, Knight R 2009 Bacterial community variation in human body habitats across space and time. Science 326:1694–1697. doi:10.1126/science.1177486. 19892944 PMC3602444

[9] Daniel H, Gholami AM, Berry D, Desmarchelier C, Hahne H, Loh G, Mondot S, Lepage P, Rothballer M, Walker A, Bohm C, Wenning M, Wagner M, Blaut M, Schmitt-Kopplin P, Kuster B, Haller D, Clavel T 2014 High-fat diet alters gut microbiota physiology in mice. ISME J 8:295–308. doi:10.1038/ismej.2013.155. 24030595 PMC3906816

[10] Schulz MD, Atay C, Heringer J, Romrig FK, Schwitalla S, Aydin B, Ziegler PK, Varga J, Reindl W, Pommerenke C, Salinas-Riester G, Bock A, Alpert C, Blaut M, Polson SC, Brandl L, Kirchner T, Greten FR, Polson SW, Arkan MC 2014 High-fat-diet-mediated dysbiosis promotes intestinal carcinogenesis independently of obesity. Nature 514:508–512. doi:10.1038/nature13398. 25174708 PMC4233209

[11] Walker A, Pfitzner B, Neschen S, Kahle M, Harir M, Lucio M, Moritz F, Tziotis D, Witting M, Rothballer M, Engel M, Schmid M, Endesfelder D, Klingenspor M, Rattei T, Castell WZ, de Angelis MH, Hartmann A, Schmitt-Kopplin P 2014 Distinct signatures of host-microbial meta-metabolome and gut microbiome in two C57BL/6 strains under high-fat diet. ISME J 8:2380–2396. doi:10.1038/ismej.2014.79. 24906017 PMC4260703

[12] Willing BP, Dicksved J, Halfvarson J, Andersson AF, Lucio M, Zheng Z, Jarnerot G, Tysk C, Jansson JK, Engstrand L 2010 A pyrosequencing study in twins shows that gastrointestinal microbial profiles vary with inflammatory bowel disease phenotypes. Gastroenterology 139:1844–1854.e1. doi:10.1053/j.gastro.2010.08.049. 20816835

[13] Arrieta MC, CHILD Study Investigators, Stiemsma LT, Dimitriu PA, Thorson L, Russell S, Yurist-Doutsch S, Kuzeljevic B, Gold MJ, Britton HM, Lefebvre DL, Subbarao P, Mandhane P, Becker A, McNagny KM, Sears MR, Kollmann T, Mohn WW, Turvey SE, Brett Finlay B 2015 Early infancy microbial and metabolic alterations affect risk of childhood asthma. Sci Transl Med 7:307ra152. doi:10.1126/scitranslmed.aab2271. 26424567

[14] Relman DA 2012 The human microbiome: ecosystem resilience and health. Nutr Rev 70 (Suppl 1):S2–S9. doi:10.1111/j.1753-4887.2012.00489.x. 22861804 PMC3422777

[15] Fujimura KE, Slusher NA, Cabana MD, Lynch SV 2010 Role of the gut microbiota in defining human health. Expert Rev Anti Infect Ther 8:435–454. doi:10.1586/eri.10.14. 20377338 PMC2881665

[16] Minot S, Sinha R, Chen J, Li H, Keilbaugh SA, Wu GD, Lewis JD, Bushman FD 2011 The human gut virome: inter-individual variation and dynamic response to diet. Genome Res 21:1616–1625. doi:10.1101/gr.122705.111. 21880779 PMC3202279

[17] Pride DT, Salzman J, Haynes M, Rohwer F, Davis-Long C, White RA 3rd, Loomer P, Armitage GC, Relman DA 2012 Evidence of a robust resident bacteriophage population revealed through analysis of the human salivary virome. ISME J 6:915–926. doi:10.1038/ismej.2011.169. 22158393 PMC3329113

[18] Willner D, Furlan M, Schmieder R, Grasis JA, Pride DT, Relman DA, Angly FE, McDole T, Mariella RP Jr, Rohwer F, Haynes M 2011 Metagenomic detection of phage-encoded platelet-binding factors in the human oral cavity. Proc Natl Acad Sci U S A 108 (Suppl 1):4547–4553. doi:10.1073/pnas.1000089107. 20547834 PMC3063595

[19] Breitbart M, Hewson I, Felts B, Mahaffy JM, Nulton J, Salamon P, Rohwer F 2003 Metagenomic analyses of an uncultured viral community from human feces. J Bacteriol 185:6220–6223. doi:10.1128/jb.185.20.6220-6223.2003. 14526037 PMC225035

[20] Reyes A, Haynes M, Hanson N, Angly FE, Heath AC, Rohwer F, Gordon JI 2010 Viruses in the faecal microbiota of monozygotic twins and their mothers. Nature 466:334–338. doi:10.1038/nature09199. 20631792 PMC2919852

[21] Kim MS, Bae JW 2016 Spatial disturbances in altered mucosal and luminal gut viromes of diet-induced obese mice. Environ Microbiol 18:1498–1510. doi:10.1111/1462-2920.13182. 26690305

[22] Abeles SR, Pride DT 2014 Molecular bases and role of viruses in the human microbiome. J Mol Biol 426:3892–3906. doi:10.1016/j.jmb.2014.07.002. 25020228 PMC7172398

[23] Modi SR, Lee HH, Spina CS, Collins JJ 2013 Antibiotic treatment expands the resistance reservoir and ecological network of the phage metagenome. Nature 499:219–222. doi:10.1038/nature12212. 23748443 PMC3710538

[24] Reyes A, Wu M, McNulty NP, Rohwer FL, Gordon JI 2013 Gnotobiotic mouse model of phage-bacterial host dynamics in the human gut. Proc Natl Acad Sci U S A 110:20236–20241. doi:10.1073/pnas.1319470110. 24259713 PMC3864308

[25] Barr JJ, Auro R, Furlan M, Whiteson KL, Erb ML, Pogliano J, Stotland A, Wolkowicz R, Cutting AS, Doran KS, Salamon P, Youle M, Rohwer F 2013 Bacteriophage adhering to mucus provide a non-host-derived immunity. Proc Natl Acad Sci U S A 110:10771–10776. doi:10.1073/pnas.1305923110. 23690590 PMC3696810

[26] Chibani-Chennoufi S, Bruttin A, Dillmann M-L, Brüssow H 2004 Phage-host interaction: an ecological perspective. J Bacteriol 186:3677–3686. doi:10.1128/JB.186.12.3677-3686.2004. 15175280 PMC419959

[27] Kless C, Muller VM, Schuppel VL, Lichtenegger M, Rychlik M, Daniel H, Klingenspor M, Haller D 2015 Diet-induced obesity causes metabolic impairment independent of alterations in gut barrier integrity. Mol Nutr Food Res 59:968–978. doi:10.1002/mnfr.201400840. 25676872

[28] Muller VM, Zietek T, Rohm F, Fiamoncini J, Lagkouvardos I, Haller D, Clavel T, Daniel H 2016 Gut barrier impairment by high-fat diet in mice depends on housing conditions. Mol Nutr Food Res 60:897–908. doi:10.1002/mnfr.201500775. 26679432

[29] Cani PD, Bibiloni R, Knauf C, Waget A, Neyrinck AM, Delzenne NM, Burcelin R 2008 Changes in gut microbiota control metabolic endotoxemia-induced inflammation in high-fat diet-induced obesity and diabetes in mice. Diabetes 57:1470–1481. doi:10.2337/db07-1403. 18305141

[30] Vrieze A, Van Nood E, Holleman F, Salojarvi J, Kootte RS, Bartelsman JF, Dallinga-Thie GM, Ackermans MT, Serlie MJ, Oozeer R, Derrien M, Druesne A, Van Hylckama Vlieg JE, Bloks VW, Groen AK, Heilig HG, Zoetendal EG, Stroes ES, de Vos WM, Hoekstra JB, Nieuwdorp M 2012 Transfer of intestinal microbiota from lean donors increases insulin sensitivity in individuals with metabolic syndrome. Gastroenterology 143:913–916.e7. doi:10.1053/j.gastro.2012.06.031. 22728514

[31] Cani PD, Amar J, Iglesias MA, Poggi M, Knauf C, Bastelica D, Neyrinck AM, Fava F, Tuohy KM, Chabo C, Waget A, Delmee E, Cousin B, Sulpice T, Chamontin B, Ferrieres J, Tanti JF, Gibson GR, Casteilla L, Delzenne NM, Alessi MC, Burcelin R 2007 Metabolic endotoxemia initiates obesity and insulin resistance. Diabetes 56:1761–1772. doi:10.2337/db06-1491. 17456850

[32] Wiśniewski JR, Friedrich A, Keller T, Mann M, Koepsell H 2015 The impact of high-fat diet on metabolism and immune defense in small intestine mucosa. J Proteome Res 14:353–365. doi:10.1021/pr500833v. 25285821

[33] Cho I, Yamanishi S, Cox L, Methe BA, Zavadil J, Li K, Gao Z, Mahana D, Raju K, Teitler I, Li H, Alekseyenko AV, Blaser MJ 2012 Antibiotics in early life alter the murine colonic microbiome and adiposity. Nature 488:621–626. doi:10.1038/nature11400. 22914093 PMC3553221

[34] Mahana D, Trent CM, Kurtz ZD, Bokulich NA, Battaglia T, Chung J, Muller CL, Li H, Bonneau RA, Blaser MJ 2016 Antibiotic perturbation of the murine gut microbiome enhances the adiposity, insulin resistance, and liver disease associated with high-fat diet. Genome Med 8:48. doi:10.1186/s13073-016-0297-9. 27124954 PMC4847194

[35] Cox LM, Yamanishi S, Sohn J, Alekseyenko AV, Leung JM, Cho I, Kim SG, Li H, Gao Z, Mahana D, Zarate Rodriguez JG, Rogers AB, Robine N, Loke P, Blaser MJ 2014 Altering the intestinal microbiota during a critical developmental window has lasting metabolic consequences. Cell 158:705–721. doi:10.1016/j.cell.2014.05.052. 25126780 PMC4134513

[36] Schulfer AF, Schluter J, Zhang Y, Brown Q, Pathmasiri W, McRitchie S, Sumner S, Li H, Xavier JB, Blaser MJ 2019 The impact of early-life sub-therapeutic antibiotic treatment (STAT) on excessive weight is robust despite transfer of intestinal microbes. ISME J 13:1280–1292. doi:10.1038/s41396-019-0349-4. 30651608 PMC6474226

[37] Rothberg JM, Hinz W, Rearick TM, Schultz J, Mileski W, Davey M, Leamon JH, Johnson K, Milgrew MJ, Edwards M, Hoon J, Simons JF, Marran D, Myers JW, Davidson JF, Branting A, Nobile JR, Puc BP, Light D, Clark TA, Huber M, Branciforte JT, Stoner IB, Cawley SE, Lyons M, Fu Y, Homer N, Sedova M, Miao X, Reed B, Sabina J, Feierstein E, Schorn M, Alanjary M, Dimalanta E, Dressman D, Kasinskas R, Sokolsky T, Fidanza JA, Namsaraev E, McKernan KJ, Williams A, Roth GT, Bustillo J 2011 An integrated semiconductor device enabling non-optical genome sequencing. Nature 475:348–352. doi:10.1038/nature10242. 21776081

[38] Zhang C, Zhang M, Pang X, Zhao Y, Wang L, Zhao L 2012 Structural resilience of the gut microbiota in adult mice under high-fat dietary perturbations. ISME J 6:1848–1857. doi:10.1038/ismej.2012.27. 22495068 PMC3446802

[39] Abeles SR, Robles-Sikisaka R, Ly M, Lum AG, Salzman J, Boehm TK, Pride DT 2014 Human oral viruses are personal, persistent and gender-consistent. ISME J 8:1753–1767. doi:10.1038/ismej.2014.31. 24646696 PMC4139723

[40] Robles-Sikisaka R, Ly M, Boehm T, Naidu M, Salzman J, Pride DT 2013 Association between living environment and human oral viral ecology. ISME J 7:1710–1724. doi:10.1038/ismej.2013.63. 23598790 PMC3749502

[41] David LA, Maurice CF, Carmody RN, Gootenberg DB, Button JE, Wolfe BE, Ling AV, Devlin AS, Varma Y, Fischbach MA, Biddinger SB, Dutton RJ, Turnbaugh PJ 2014 Diet rapidly and reproducibly alters the human gut microbiome. Nature 505:559–563. doi:10.1038/nature12820. 24336217 PMC3957428

[42] Singh RK, Chang HW, Yan D, Lee KM, Ucmak D, Wong K, Abrouk M, Farahnik B, Nakamura M, Zhu TH, Bhutani T, Liao W 2017 Influence of diet on the gut microbiome and implications for human health. J Transl Med 15:73. doi:10.1186/s12967-017-1175-y. 28388917 PMC5385025

[43] Wichels A, Biel SS, Gelderblom HR, Brinkhoff T, Muyzer G, Schutt C 1998 Bacteriophage diversity in the North Sea. Appl Environ Microbiol 64:4128–4133. doi:10.1128/AEM.64.11.4128-4133.1998. 9797256 PMC106618

[44] Sullivan MB, Waterbury JB, Chisholm SW 2003 Cyanophages infecting the oceanic cyanobacterium Prochlorococcus. Nature 424:1047–1051. doi:10.1038/nature01929. 12944965

[45] Manrique P, Dills M, Young M 2017 The human gut phage community and its implications for health and disease. Viruses 9:141. doi:10.3390/v9060141. PMC5490818 28594392

[46] Furuse K, Osawa S, Kawashiro J, Tanaka R, Ozawa A, Sawamura S, Yanagawa Y, Nagao T, Watanabe I 1983 Bacteriophage distribution in human faeces: continuous survey of healthy subjects and patients with internal and leukaemic diseases. J Gen Virol 64:2039–2043. doi:10.1099/0022-1317-64-9-2039. 6886680

[47] Lepage P, Colombet J, Marteau P, Sime-Ngando T, Doré J, Leclerc M 2008 Dysbiosis in inflammatory bowel disease: a role for bacteriophages? Gut 57:424–425. doi:10.1136/gut.2007.134668. 18268057

[48] Shkoporov A, Clooney AG, Sutton TD, Ryan FJ, Daly KM, Nolan JA, McDonnell SA, Khokhlova EV, Draper LA, Forde A, Guerin E, Velayudhan V, Ross PR, Hill C 2019 The human gut virome is highly diverse, stable and individual-specific. Cell Host Microbe 26:448–449. doi:10.1016/j.chom.2019.09.009. 31600503

[49] Krupovic M, Forterre P 2011 Microviridae goes temperate: microvirus-related proviruses reside in the genomes of Bacteroidetes. PLoS One 6:e19893. doi:10.1371/journal.pone.0019893. 21572966 PMC3091885

[50] Le Chatelier E, MetaHIT consortium, Nielsen T, Qin J, Prifti E, Hildebrand F, Falony G, Almeida M, Arumugam M, Batto JM, Kennedy S, Leonard P, Li J, Burgdorf K, Grarup N, Jorgensen T, Brandslund I, Nielsen HB, Juncker AS, Bertalan M, Levenez F, Pons N, Rasmussen S, Sunagawa S, Tap J, Tims S, Zoetendal EG, Brunak S, Clement K, Dore J, Kleerebezem M, Kristiansen K, Renault P, Sicheritz-Ponten T, de Vos WM, Zucker JD, Raes J, Hansen T, Bork P, Wang J, Ehrlich SD, Pedersen O 2013 Richness of human gut microbiome correlates with metabolic markers. Nature 500:541–546. doi:10.1038/nature12506. 23985870

[51] Cotillard A, ANR MicroObes consortium, Kennedy SP, Kong LC, Prifti E, Pons N, Le Chatelier E, Almeida M, Quinquis B, Levenez F, Galleron N, Gougis S, Rizkalla S, Batto JM, Renault P, Dore J, Zucker JD, Clement K, Ehrlich SD 2013 Dietary intervention impact on gut microbial gene richness. Nature 500:585–588. doi:10.1038/nature12480. 23985875

[52] Turnbaugh PJ, Hamady M, Yatsunenko T, Cantarel BL, Duncan A, Ley RE, Sogin ML, Jones WJ, Roe BA, Affourtit JP, Egholm M, Henrissat B, Heath AC, Knight R, Gordon JI 2009 A core gut microbiome in obese and lean twins. Nature 457:480–484. doi:10.1038/nature07540. 19043404 PMC2677729

[53] Abeles SR, Ly M, Santiago-Rodriguez TM, Pride DT 2015 Effects of long term antibiotic therapy on human oral and fecal viromes. PLoS One 10:e0134941. doi:10.1371/journal.pone.0134941. 26309137 PMC4550281

[54] La Scola B, Marrie TJ, Auffray J-P, Raoult D 2005 Mimivirus in pneumonia patients. Emerg Infect Dis 11:449–452. doi:10.3201/eid1103.040538. 15757563 PMC3298252

[55] Vincent A, La Scola B, Forel J-M, Pauly V, Raoult D, Papazian L 2009 Clinical significance of a positive serology for mimivirus in patients presenting a suspicion of ventilator-associated pneumonia. Crit Care Med 37:111–118. doi:10.1097/CCM.0b013e318192fa8b. 19050618

[56] Yolken RH, Jones-Brando L, Dunigan DD, Kannan G, Dickerson F, Severance E, Sabunciyan S, Talbot CC, Prandovszky E, Gurnon JR, Agarkova IV, Leister F, Gressitt KL, Chen O, Deuber B, Ma F, Pletnikov MV, Van Etten JL 2014 Chlorovirus ATCV-1 is part of the human oropharyngeal virome and is associated with changes in cognitive functions in humans and mice. Proc Natl Acad Sci U S A 111:16106–16111. doi:10.1073/pnas.1418895111. 25349393 PMC4234575

[57] Sun S, La Scola B, Bowman VD, Ryan CM, Whitelegge JP, Raoult D, Rossmann MG 2010 Structural studies of the Sputnik virophage. J Virol 84:894–897. doi:10.1128/JVI.01957-09. 19889775 PMC2798384

[58] La Scola B, Desnues C, Pagnier I, Robert C, Barrassi L, Fournous G, Merchat M, Suzan-Monti M, Forterre P, Koonin E, Raoult D 2008 The virophage as a unique parasite of the giant mimivirus. Nature 455:100–104. doi:10.1038/nature07218. 18690211

[59] Williams SH, Che X, Garcia JA, Klena JD, Lee B, Muller D, Ulrich W, Corrigan RM, Nichol S, Jain K, Lipkin WI 2018 Viral diversity of house mice in New York City. mBio 9:e01354-17. doi:10.1128/mBio.01354-17. 29666290 PMC5904411

[60] Howe A, Ringus DL, Williams RJ, Choo ZN, Greenwald SM, Owens SM, Coleman ML, Meyer F, Chang EB 2016 Divergent responses of viral and bacterial communities in the gut microbiome to dietary disturbances in mice. ISME J 10:1217–1227. doi:10.1038/ismej.2015.183. 26473721 PMC5029215

[61] Vital M, Harkema JR, Rizzo M, Tiedje J, Brandenberger C 2015 Alterations of the murine gut microbiome with age and allergic airway disease. J Immunol Res 2015:892568. doi:10.1155/2015/892568. 26090504 PMC4451525

[62] d’Humières C, Touchon M, Dion S, Cury J, Ghozlane A, Garcia-Garcera M, Bouchier C, Ma L, Denamur E, Rocha EP 2019 A simple, reproducible and cost-effective procedure to analyse gut phageome: from phage isolation to bioinformatic approach. Sci Rep 9:1–13. doi:10.1038/s41598-019-47656-w. 31383878 PMC6683287

[63] Kim K-H, Bae J-W 2011 Amplification methods bias metagenomic libraries of uncultured single-stranded and double-stranded DNA viruses. Appl Environ Microbiol 77:7663–7668. doi:10.1128/AEM.00289-11. 21926223 PMC3209148

[64] Franklin CL, Ericsson AC 2017 Microbiota and reproducibility of rodent models. Lab Anim (NY) 46:114–122. doi:10.1038/laban.1222. 28328896 PMC5762113

[65] Murphy FA, Fauquet CM, Bishop DHL, Ghabrial SA, Jarvis AW, Martelli GP, Mayo MA, Summers MD (ed). 1995 Virus taxonomy: sixth report of the International Committee on Taxonomy of Viruses. Springer-Verlag, New York, NY.

[66] Breitbart M, Salamon P, Andresen B, Mahaffy JM, Segall AM, Mead D, Azam F, Rohwer F 2002 Genomic analysis of uncultured marine viral communities. Proc Natl Acad Sci U S A 99:14250–14255. doi:10.1073/pnas.202488399. 12384570 PMC137870

[67] Caporaso JG, Kuczynski J, Stombaugh J, Bittinger K, Bushman FD, Costello EK, Fierer N, Pena AG, Goodrich JK, Gordon JI, Huttley GA, Kelley ST, Knights D, Koenig JE, Ley RE, Lozupone CA, McDonald D, Muegge BD, Pirrung M, Reeder J, Sevinsky JR, Turnbaugh PJ, Walters WA, Widmann J, Yatsunenko T, Zaneveld J, Knight R 2010 QIIME allows analysis of high-throughput community sequencing data. Nat Methods 7:335–336. doi:10.1038/nmeth.f.303. 20383131 PMC3156573

[68] Santiago-Rodriguez TM, Ly M, Bonilla N, Pride DT 2015 The human urine virome in association with urinary tract infections. Front Microbiol 6:14. doi:10.3389/fmicb.2015.00014. 25667584 PMC4304238

[69] Santiago-Rodriguez TM, Ly M, Daigneault MC, Brown IH, McDonald JA, Bonilla N, Vercoe EA, Pride DT 2015 Chemostat culture systems support diverse bacteriophage communities from human feces. Microbiome 3:58. doi:10.1186/s40168-015-0124-3. 26549756 PMC4638026

[70] Wickham H 2009 ggplot2: elegant graphics for data analysis. Springer Science & Business Media, Berlin, Germany.

[71] Ly M, Abeles SR, Boehm TK, Robles-Sikisaka R, Naidu M, Santiago-Rodriguez T, Pride DT 2014 Altered oral viral ecology in association with periodontal disease. mBio 5:e01133-14. doi:10.1128/mBio.01133-14. 24846382 PMC4030452

[72] Whiteley AS, Jenkins S, Waite I, Kresoje N, Payne H, Mullan B, Allcock R, O’Donnell A 2012 Microbial 16S rRNA Ion Tag and community metagenome sequencing using the Ion Torrent (PGM) platform. J Microbiol Methods 91:80–88. doi:10.1016/j.mimet.2012.07.008. 22849830

[73] Ewing B, Green P 1998 Base-calling of automated sequencer traces using phred. II. Error probabilities. Genome Res 8:186–194. doi:10.1101/gr.8.3.186. 9521922

[74] DeSantis TZ, Hugenholtz P, Larsen N, Rojas M, Brodie EL, Keller K, Huber T, Dalevi D, Hu P, Andersen GL 2006 Greengenes, a chimera-checked 16S rRNA gene database and workbench compatible with ARB. Appl Environ Microbiol 72:5069–5072. doi:10.1128/AEM.03006-05. 16820507 PMC1489311

[75] Lozupone C, Hamady M, Knight R 2006 UniFrac—an online tool for comparing microbial community diversity in a phylogenetic context. BMC Bioinformatics 7:371. doi:10.1186/1471-2105-7-371. 16893466 PMC1564154

[76] Gotelli NJ, Colwell RK 2001 Quantifying biodiversity: procedures and pitfalls in the measurement and comparison of species richness. Ecol Lett 4:379–391. doi:10.1046/j.1461-0248.2001.00230.x.

