# Supplementary figures and images for: Fecal Viral Community Responses to High-Fat Diet in Mice
Source: mSphere. 2020 Feb 26;5(1):e00833-19. doi: 10.1128/mSphere.00833-19 (PMC7045389; doi:10.1128/mSphere.00833-19)

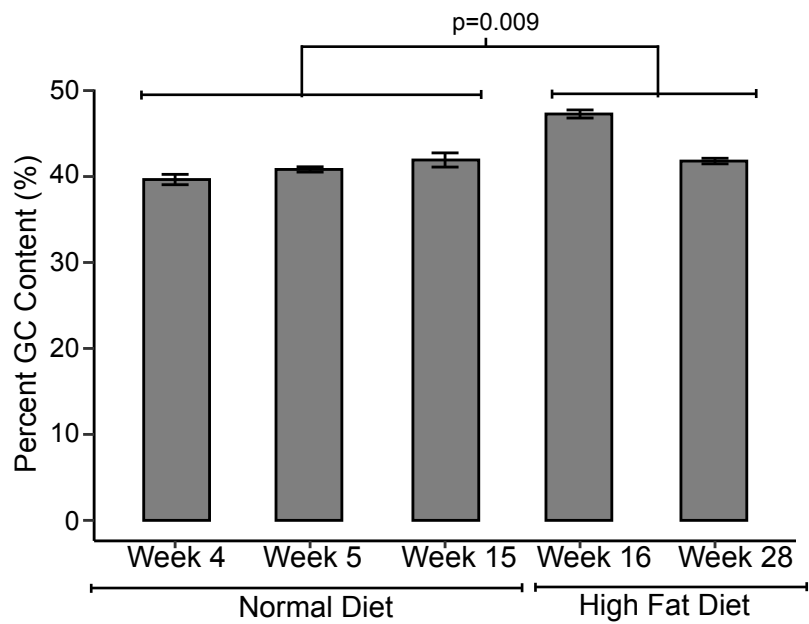

Supplement: FIG S1 [file mSphere.00833-19-sf001.pdf]

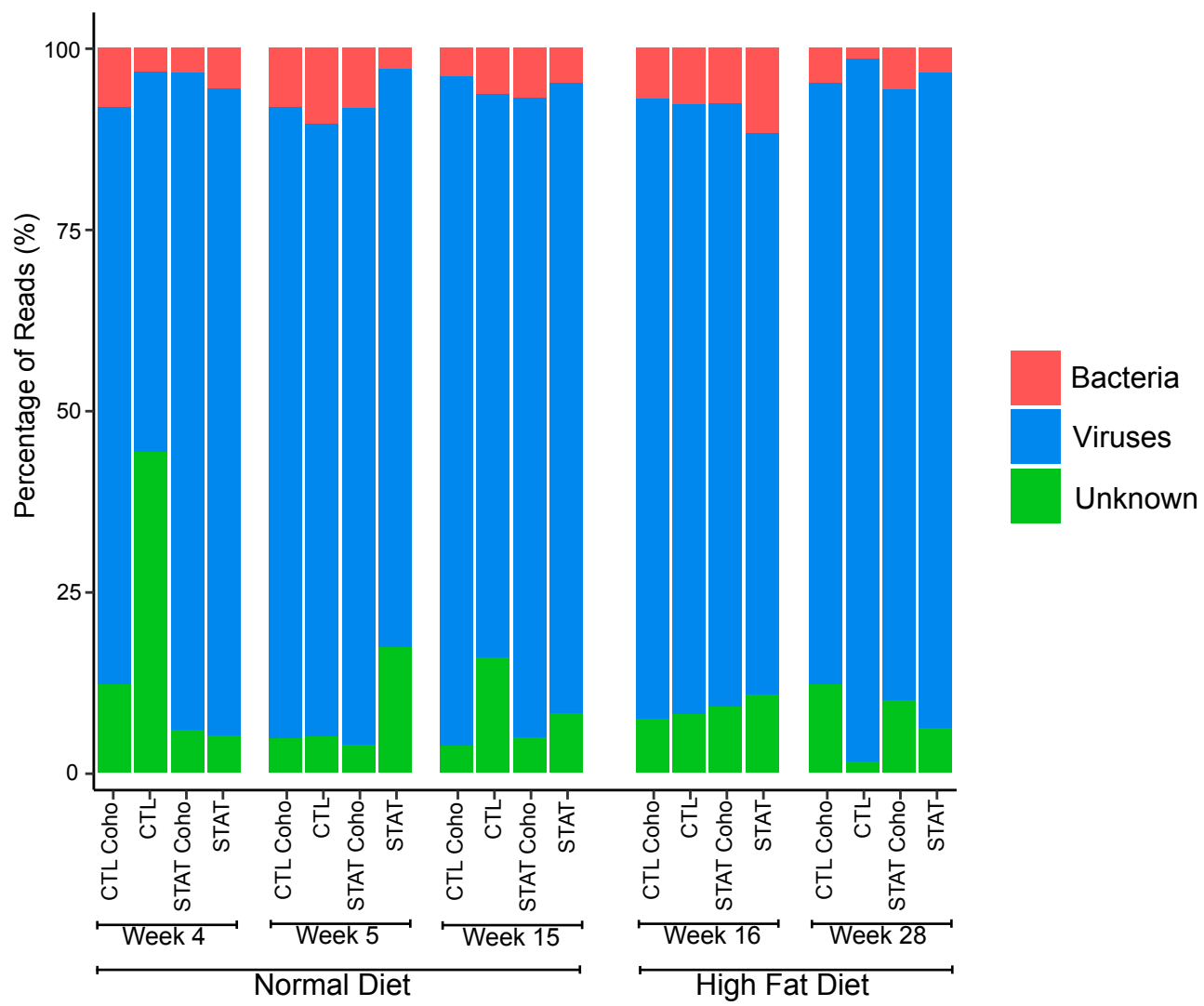

Supplement: FIG S2 [file mSphere.00833-19-sf002.pdf]

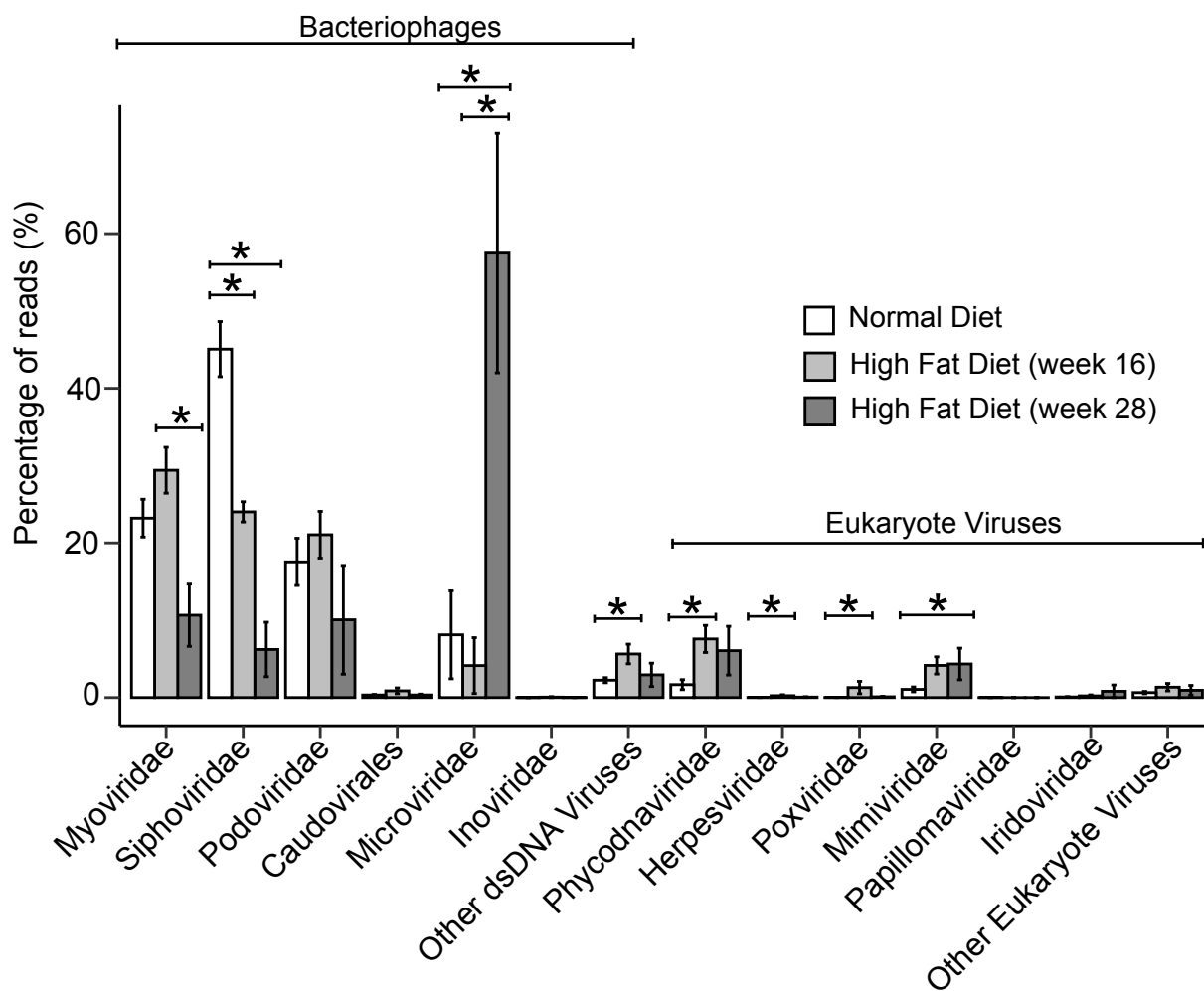

Supplement: FIG S3 [file mSphere.00833-19-sf003.pdf]

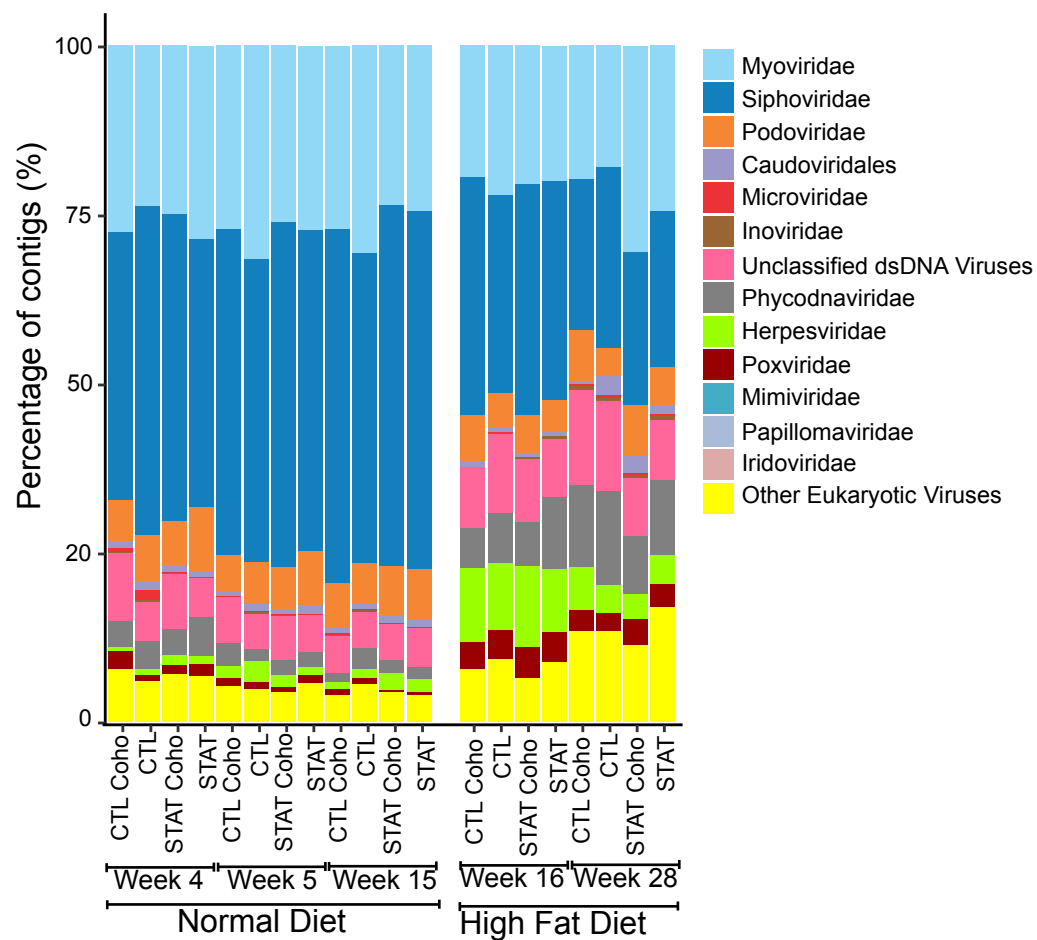

Supplement: FIG S4 [file mSphere.00833-19-sf004.pdf]

A.

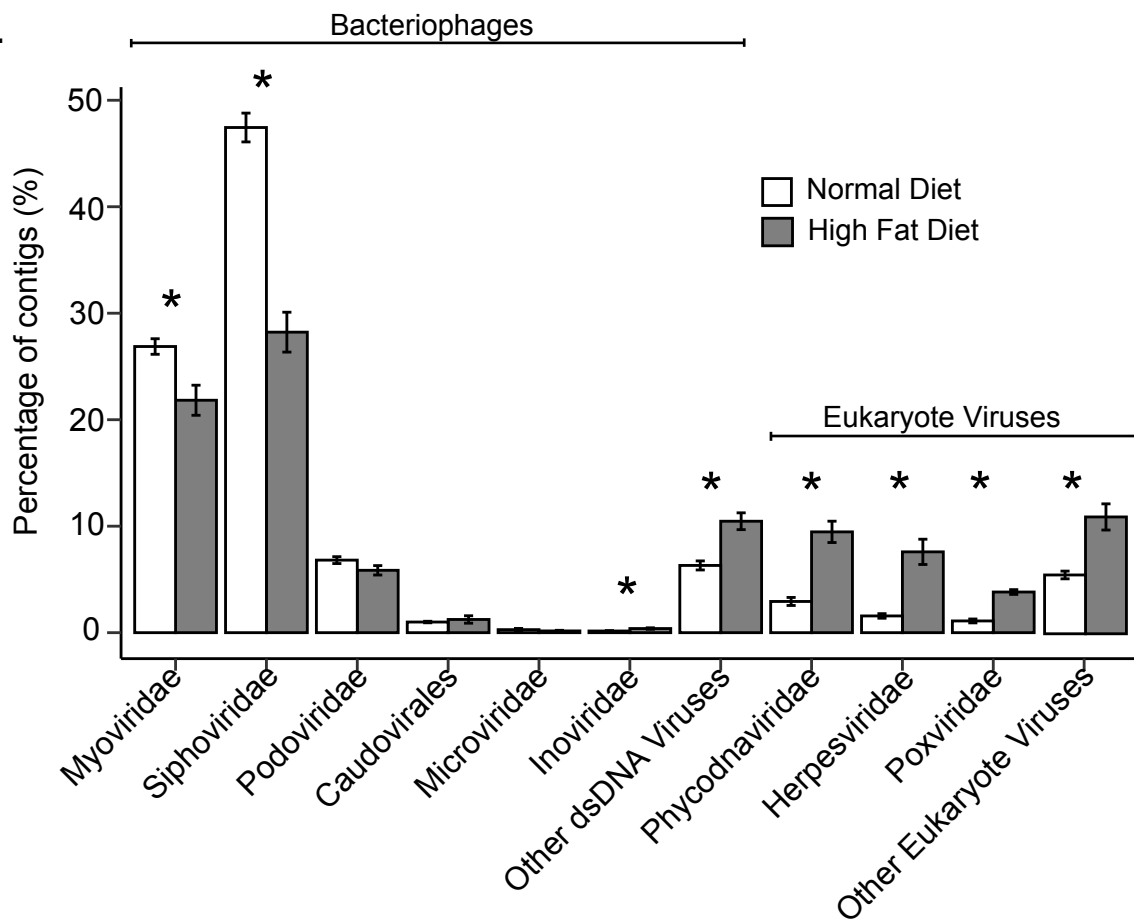

B.

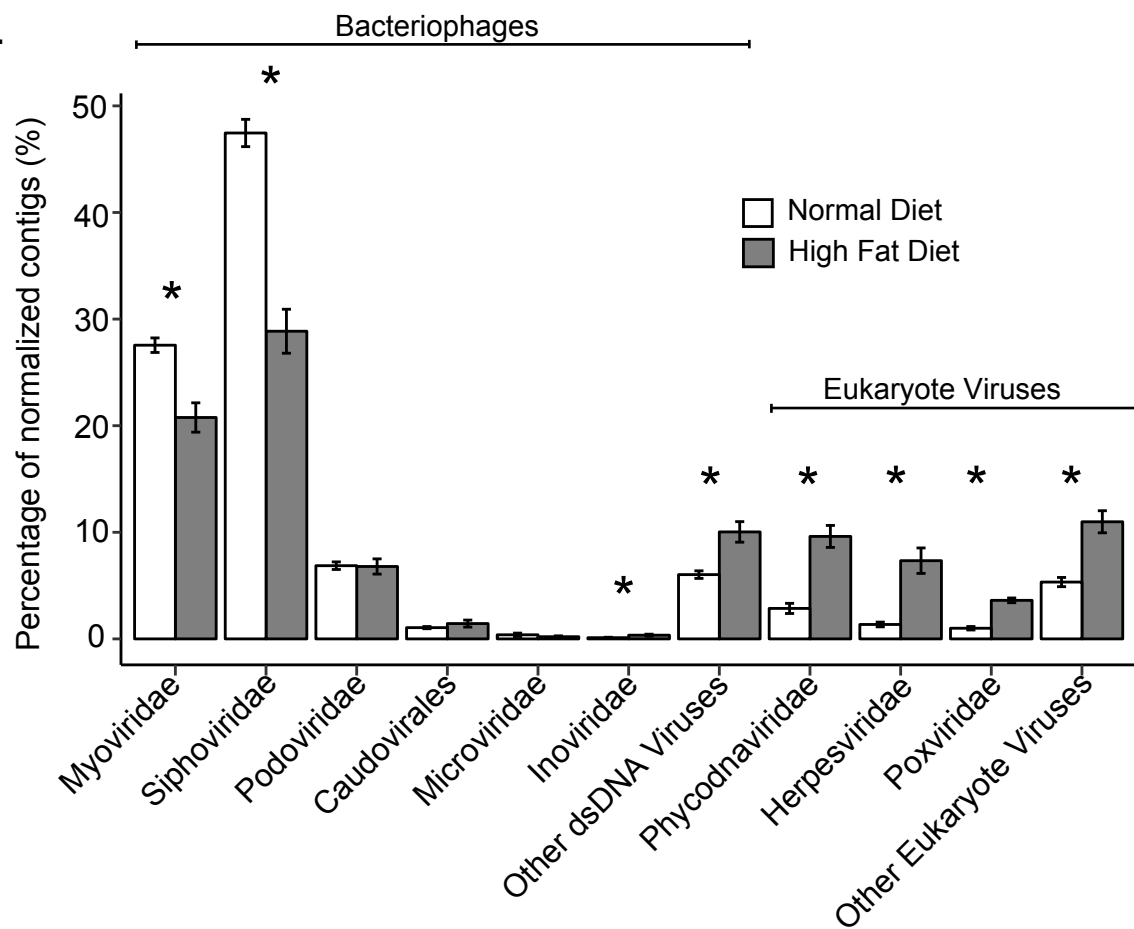

Supplement: FIG S5 [file mSphere.00833-19-sf005.pdf]

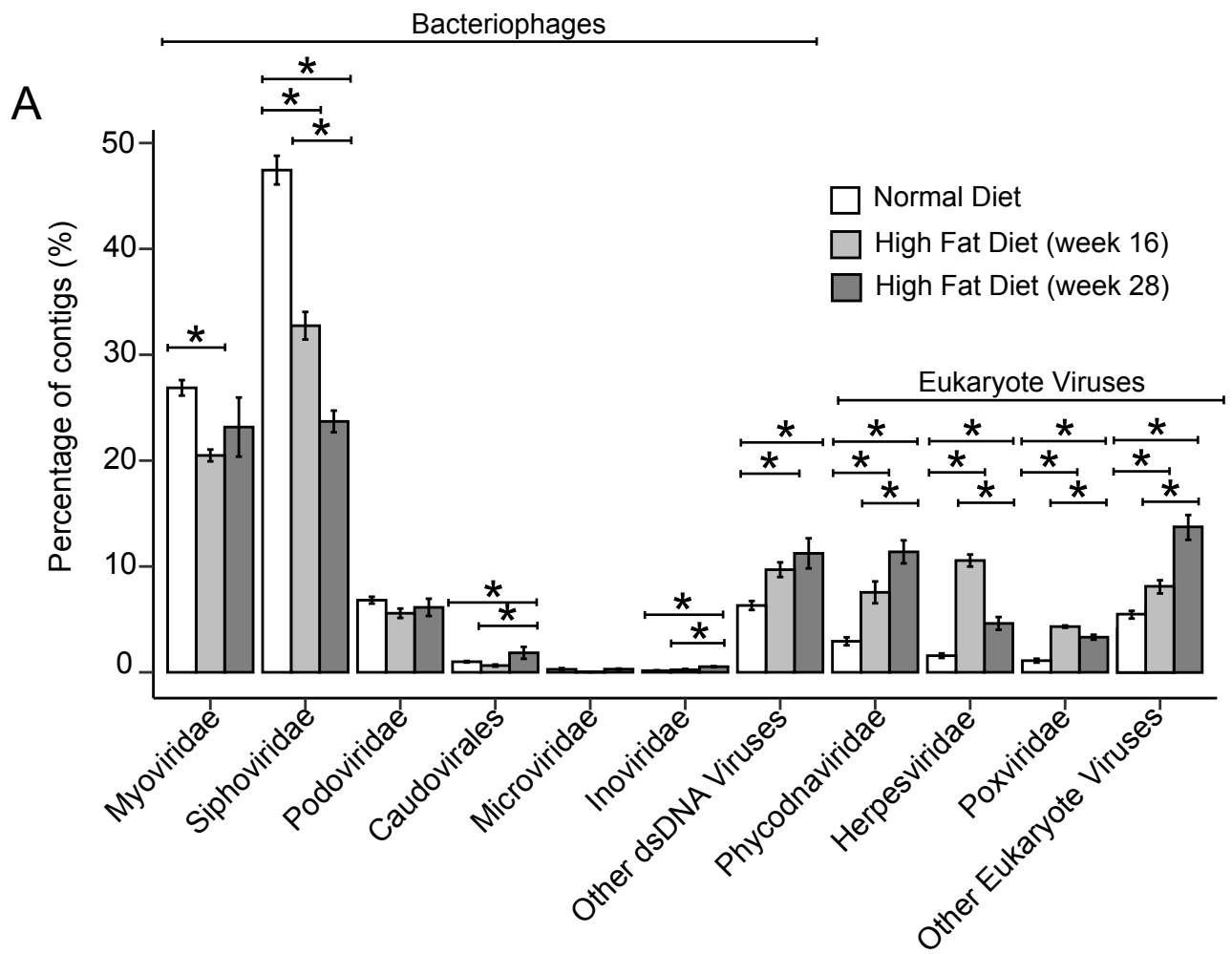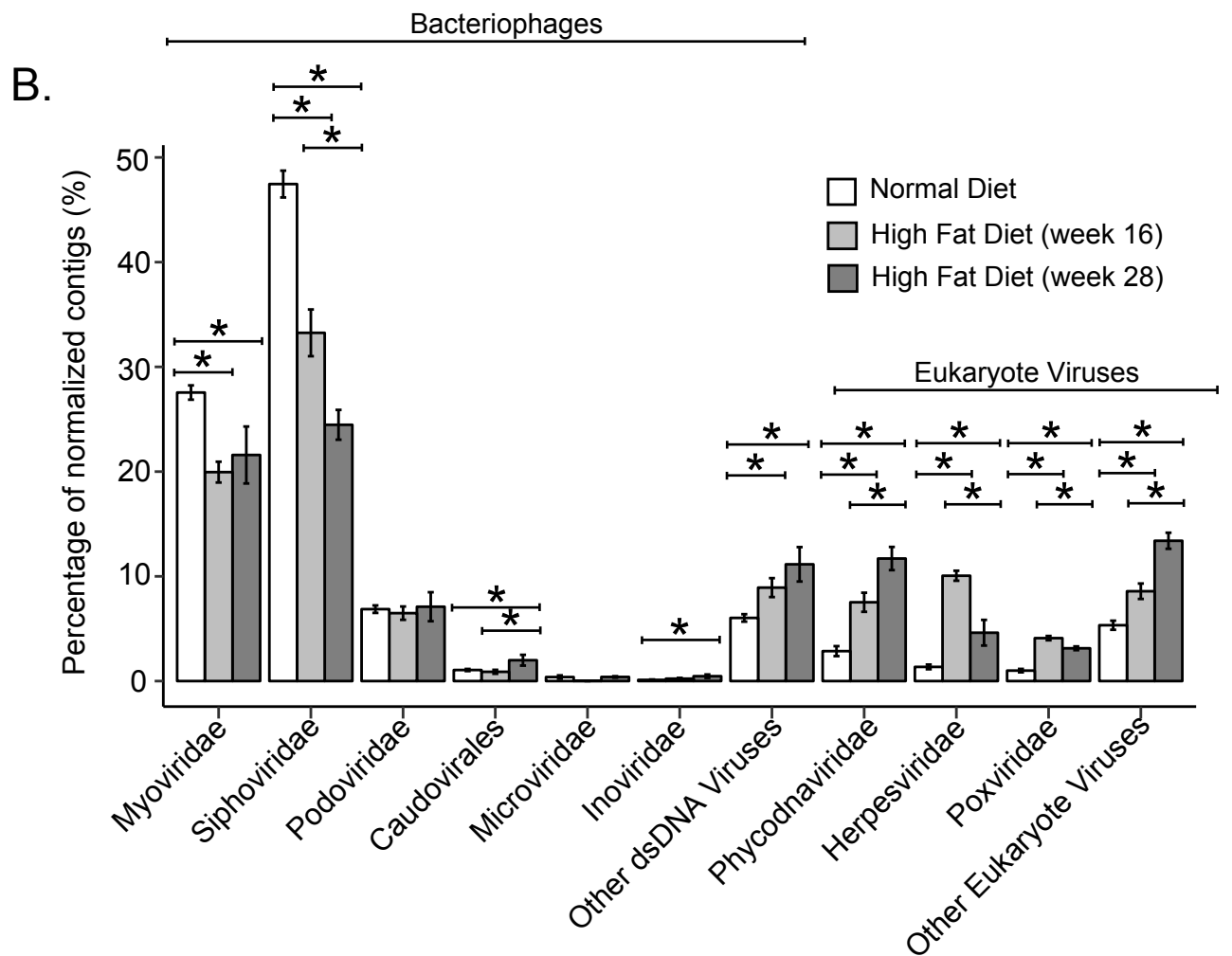

Supplement: FIG S6 [file mSphere.00833-19-sf006.pdf]
