# Supplementary material for: Fecal Viral Community Responses to High-Fat Diet in Mice
Source: mSphere. 2020 Feb 26;5(1):e00833-19. doi: 10.1128/mSphere.00833-19 (PMC7045389; doi:10.1128/mSphere.00833-19)
Supplement: TABLE S1 [file mSphere.00833-19-st001.docx]

Supplemental Table 1. Virome reads and assembly statistics.

| **Sample^a^** | **# reads** | **Mean length (nt)** | **GC %** | **% Reads assembled into contigs** |  | **# contigs** | **Mean length (nt)** | **GC %** |
| --- | --- | --- | --- | --- | --- | --- | --- | --- |
| 1CTL | 363558 | 233 | 38.8 | 94.9 |  | 1665 | 865 | 38.11 |
| 2CTL | 625635 | 213 | 40.5 | 94.3 |  | 3631 | 919 | 39.80 |
| 3CTL | 394219 | 211 | 40.1 | 95.2 |  | 3366 | 927 | 39.43 |
| 4CTL | 465148 | 197 | 47.5 | 89.8 |  | 7618 | 869 | 46.69 |
| 5CTL | 440630 | 206 | 41.8 | 97.9 |  | 1358 | 926 | 41.07 |
|  |  |  |  |  |  |  |  |  |
| 1STAT | 519311 | 214 | 40.0 | 94.1 |  | 3237 | 1103 | 40.47 |
| 2STAT | 618532 | 218 | 40.2 | 96.9 |  | 3294 | 958 | 40.96 |
| 3STAT | 364236 | 221 | 43.0 | 94.4 |  | 4024 | 914 | 42.90 |
| 4STAT | 564098 | 218 | 46.4 | 95.5 |  | 4313 | 1053 | 47.65 |
| 5STAT | 479757 | 217 | 41.5 | 96.2 |  | 1813 | 1194 | 40.43 |
|  |  |  |  |  |  |  |  |  |
| 1CTL-Coho | 532505 | 229 | 38.6 | 92.1 |  | 2221 | 979 | 37.93 |
| 2CTL-Coho | 550427 | 224 | 41.0 | 94.7 |  | 4243 | 1016 | 40.33 |
| 3CTL-Coho | 636561 | 215 | 41.0 | 96.7 |  | 3926 | 815 | 40.36 |
| 4CTL-Coho | 485132 | 218 | 46.7 | 95.4 |  | 5146 | 1010 | 45.91 |
| 5CTL-Coho | 511059 | 212 | 42.7 | 95.9 |  | 2383 | 972 | 41.92 |
|  |  |  |  |  |  |  |  |  |
| 1STAT-Coho | 894287 | 215 | 41.2 | 94.0 |  | 3255 | 930 | 40.47 |
| 2STAT-Coho | 677957 | 207 | 41.6 | 95.2 |  | 4260 | 892 | 40.96 |
| 3STAT-Coho | 541767 | 221 | 43.6 | 92.9 |  | 5210 | 883 | 42.90 |
| 4STAT-Coho | 369736 | 217 | 48.5 | 94.5 |  | 4623 | 994 | 47.65 |
| 5STAT-Coho | 555813 | 215 | 41.2 | 96.5 |  | 1856 | 1149 | 40.43 |

^a^Numbers represent time points, CTL mice received control water, STAT mice received water supplemented with

penicillin, and COHO represents CTL mice that were cohoused with STAT mice, or vice versa.
